# Implementing community case management of malaria: Stakeholder insights on advancing equitable access in Kilifi County

**DOI:** 10.1371/journal.pgph.0006478

**Published:** 2026-07-06

**Authors:** Evelyn Waweru, Moses Chapa Kiti, Grace Masha, Benjamin Tsofa, Gabriele Paone, George Dzombo, Gabriele Cerini, Maria Vittoria De Vita, Gianfranco Morino, Federico Gobbi, Paolo Leoncini, Edward Chimwaga Mwamuye, Simon Chengo Masha

**Affiliations:** 1 Think Health Consultants Limited, Kilifi, Kenya; 2 KEMRI Wellcome Trust Research Programme, Kilifi, Kenya; 3 Amici del Mondo-World Friends ETS, Nairobi, Kenya; 4 Centro Salute Globale - Regione Toscana, Firenze, Italy; 5 Department of Infectious/Tropical Diseases and Microbiology, IRCCS Sacro Cuore Don Calabria, Negrar di Valpolicella, Verona, Italy; 6 Department of Clinical and Experimental Sciences, University of Brescia, Brescia, Italy; 7 Department of Health Kilifi County, Kilifi, Kenya; 8 Department of Biological Sciences, Pwani University, Kilifi, Kenya; London School of Hygiene & Tropical Medicine, UNITED KINGDOM OF GREAT BRITAIN AND NORTHERN IRELAND

## Abstract

Malaria detection in Kilifi, County, primarily relies on intermittent active surveillance at healthcare facilities. To strengthen control of uncomplicated malaria, community health promoters (CHPs) were engaged in community health education, case detection, and treatment. This study examines lessons from integrating CHPs in community case management of malaria (CCMm). We use a mixed-method, concurrent cross-sectional design, to document stakeholder experiences of CCMm within piloted Community Health Units (CHUs) in Kilifi County. Quantitative analysis reviewed routinely collected malaria data from 01/01/2025–30/06/2025. Qualitative data were obtained through in-depth interviews with county and sub-county managers (n = 5), facility in-charges (n = 10), Community Health Assistants (CHP supervisors) (n = 12); group discussions with CHPs (n = 14) and focus group discussions with community members (n = 12). Data were analyzed thematically to describe the CCMm implementation process, enablers and challenges, and perceived outcomes. Stakeholders recognized CCMm as an effective approach for expanding access to malaria testing and treatment in underserved areas. In a pilot involving 10 facilities in Kilifi County, CHPs managed 17% (1,268 of 7,568) of malaria cases. Critical enablers included integration with the Kilifi County Department of Health, which supported CHP training, supervision, and supply of rapid diagnostic tests (RDTs). Community trust and initial facility-based mentorship enhanced CHP competence and promoted acceptance of CHPs in their expanded roles. However, implementation faced challenges, including irregular antimalarial supplies, inconsistent CHP remuneration, limited logistical support, and weak integration with referral and information systems. As donor support phases out, stakeholders underscored the need for increased county government ownership, institutional capacity building, and sustainable resource allocation to ensure the continuity and scalability of CCMm interventions. Stakeholder experiences in Kilifi County underscore CCMm’s potential to enhance malaria control, while revealing systemic and resource-related challenges. Addressing these barriers is critical for optimizing equitable, community-level malaria interventions and informing policy in comparable settings.

## Introduction

Malaria remains an important global public health challenge, particularly in tropical and subtropical regions. According to the World Malaria Report for 2025, there were an estimated 282 million cases of malaria globally in 2024, with an incidence of 64 cases per 1000 population at risk. This represented an increase of 9 million cases from the previous year and a rise in incidence from 62.7 cases per 1000 population at risk in 2023. The WHO African Region continues to carry the heaviest burden of malaria, accounting for an estimated 94% of malaria cases and 95% of malaria deaths worldwide in 2024; 75% of all deaths in this region were among children aged under 5 years old [1]. The resurgence of malaria in Africa can be attributed to lack of funding for malaria control programs, coupled with poor malaria program execution, complacency with the malaria situation, challenges in community engagement and low levels of community participation [2,3].

The World Health Organization (WHO) outlines three core pillars for malaria elimination in its Global Technical Strategy 2016–2030 [4]. These include universal access to prevention including insecticide-treated nets (ITNs), diagnosis, and treatment artemisinin-based combination therapies (ACTs); accelerated elimination efforts through strengthened surveillance, targeted interventions in high-transmission areas, and certification support; and research transformation to develop novel tools (vaccines, next-gen insecticides) and optimize implementation strategies. All these are underpinned by health system strengthening and cross-border collaboration to achieve global eradication targets. Climate change, population movements, antimalarial and/or insecticide resistance and gaps in healthcare access continue to complicate malaria elimination efforts and require innovative ways of bridging the gaps [5–7].

In Kenya malaria accounts for an estimated 15 percent of outpatient consultations [8]. Malaria transmission and infection risk in Kenya are mainly determined by altitude, rainfall patterns, and temperature, leading to considerable variation in malaria prevalence by season and across geographic zones. Approximately 70% of the population is at risk of malaria, including 13 million people in endemic areas and another 19 million in highland epidemic prone and seasonal transmission areas [8]. The Kenya Malaria Indicator Survey (KMS) 2019–2023 classifies Kilifi as endemic for malaria transmission [9].

Malaria Community Case Management (CCMm) is a strategy to improve access to prompt diagnosis and treatment of uncomplicated malaria at the community level, particularly in underserved areas [10]. It relies on trained community health promoters (CHPs) to deliver essential malaria services [11]. This approach has been employed in both high and low transmission areas with different outputs, including parts of Western Kenya [12]. Reports estimate that community case management CCMm can reduce overall and malaria-specific under-five mortality by 40 and 60 percent, respectively, and severe malaria morbidity by 53 percent [13].

Against this background, our study set out to explore lessons of implementing CCMm, by engaging malaria stakeholders, including staff and CHPs linked to ten health facilities in Kilifi County, where CCMm was piloted.

### Activities carried out as part of CCMm training and support in Kilifi

The Kilifi County department of Health together with Amici del Mondo-World Friends ETS Kilifi ran 2 phases of training. The first was conducted in March - June 2024, and the second one in October - November 2024 and involved training of ICs, CHAs, CHPs and some community members (including village elders, local administration and traditional healers). Both trainings covered: malaria, CCMm, malaria prevention (Long lasting Insecticidal Nets- LLINs; IPTp, indoor residual spraying, mosquito and repellents), how to identify signs and symptoms (complicated vs uncomplicated), testing using rapid diagnostic tests (RDTs), treatment of uncomplicated malaria, recognizing the signs and symptoms of severe malaria and referral of complicated cases, and reporting on malaria indicators. Participants were also trained in reporting malaria indicators in various community and facility level documentation.

CCMm in practice was consistently aligned with the various modules of training. CCMm implementation in Kilifi is guided by the Kenya Malaria Strategy 2023–2027 [14]. Kilifi County's malaria control strategy involves several key initiatives, including the distribution of long-lasting insecticidal mosquito nets (LLINs), training community health promoters to conduct malaria testing using RDTs, treatment of uncomplicated malaria, and community-led efforts like spraying breeding grounds. Over the last two years, Kilifi County has successfully implemented a two-year Community Case Management of malaria (CCMm) project, a collaborative effort led by Amici del Mondo-World Friends ETS and funded by the Italian Agency for Development Cooperation, in partnership with the Ministry of Health. This initiative empowered Community Health Promoters (CHPs) to test and treat malaria in children aged 8 months to 5 years, significantly reducing the burden on local health facilities. The project successfully trained 100 CHPs and 55 healthcare workers in malaria case management.

Understanding factors influencing CHPs performance in these relatively new roles is essential to ensure successful implementation. Regular performance evaluations through surveys coupled with timely feedback of findings to local stakeholders can support improved malaria control efforts in endemic areas. The overall objective of the study was to gather insights and lessons from the integration of CHPs into malaria community case management (CCMm) in Kilifi County, Kenya.

## Methods

### Study design and setting

**Fig 1:**
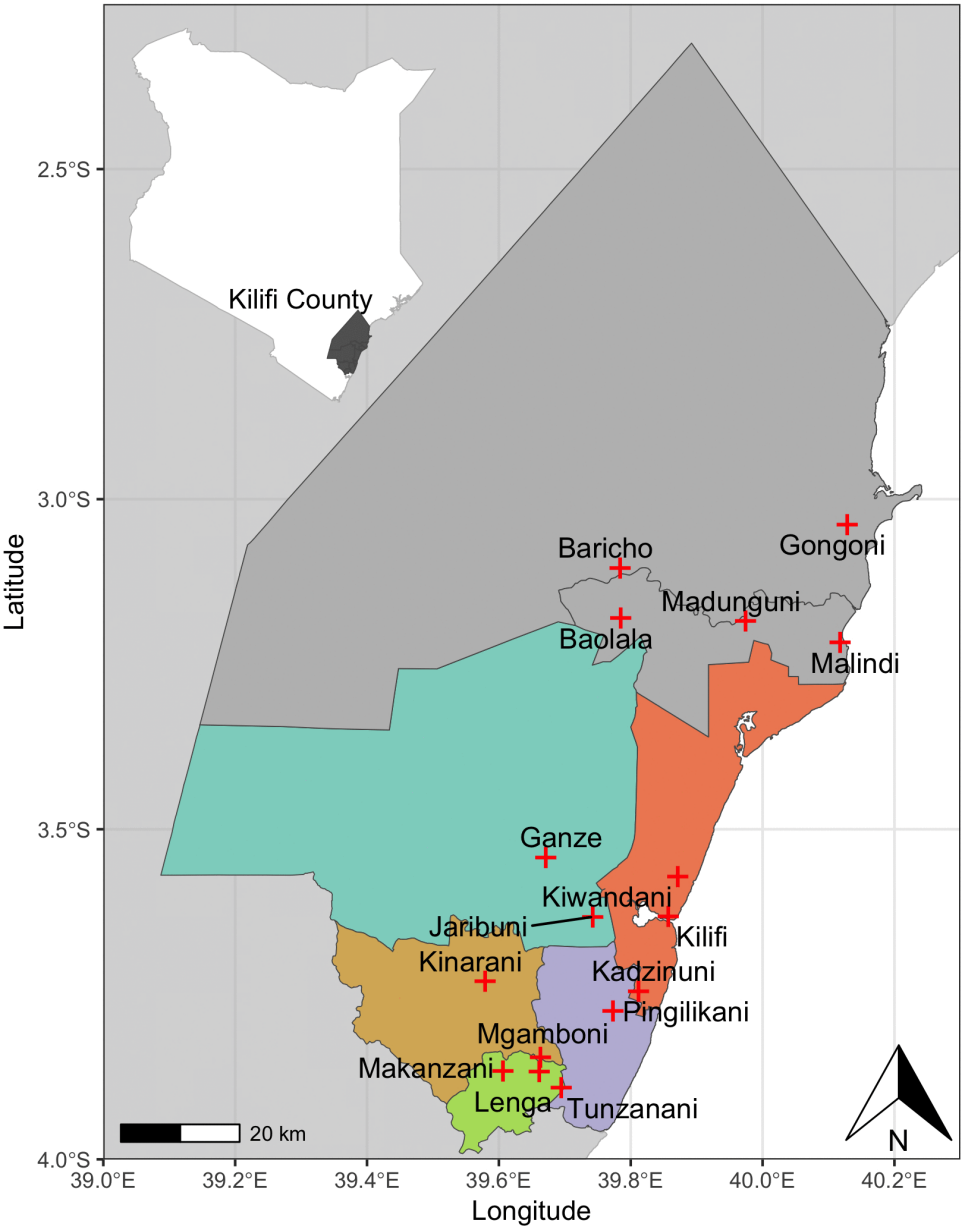
Map showing the location of fourteen study health facilities located in seven sub-counties in Kilifi. These include ten health facilities from five sub counties: Ganze (Ganze, Jaribuni), Kaloleni (Kinarani, Mgamboni), Kilifi North (Kiwandani, Kadzinuni), Kilifi South (Tunzanani, Pingilikani), and Rabai (Lenga, Makanzani) where CCMm was implemented. Data was also collected from four primary health facilities in Malindi sub-county (Madunguni, Baolala) and Magarini sub-county (Gongoni, Baricho) where CCMm was not implemented. Basemap source: GADM https://gadm.org/maps/KEN.html, CC BY 4.0 https://gadm.org/license.html.

This study employed a mixed-method, concurrent cross-sectional design, combining quantitative and qualitative approaches to document stakeholder experiences in implementing CCMm within piloted Community Health Units (CHUs) in Kilifi County. Data were collected from fourteen health facilities across all seven sub-counties in Kilifi. CCMm was implemented in ten facilities located in five sub-counties (Kilifi North, Kilifi South, Ganze, Kaloleni and Rabai). The remaining four facilities, situated in Magarini and Malindi where CCMm had not been implemented were purposively selected to qualitatively explore the existing standard for care of malaria patients in non-CCMm settings (Fig 1).

Qualitative data was collected between 01/03/2025 and 31/05/2025 while the quantitative data represented in this manuscript is from 01/01/2025–30/06/2025, congruent with the six months of implementation of the CCMm in Kilifi.

### Data collection

The study employed mixed methods including secondary quantitative data extracted from the Kenya Health Information System (KHIS), which aggregates routine programmatic data for malaria surveillance and intervention monitoring.

### Conceptual framework and development of qualitative tools

**Fig 2:**
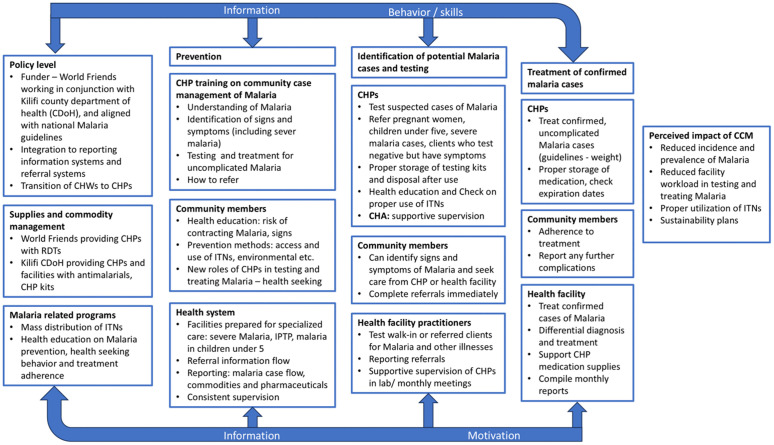
Study conceptual framework to understand the process of implementation of community case management of malaria (CCMm) in Kilifi.

The study’s conceptual framework to understand the process of implementation of CCMm in Kilifi is based on the Information – Motivation – Behavior skills (IMB) theory [15], which posits that an individual’s health behavior is predicted by their access to information, motivation, and behavioral skills to perform the behavior. In the study context we assume that community members with knowledge on malaria transmission and preventive practices will effectively use them, e.g., sleep under a treated net every night; and can easily identify signs of malaria and seek appropriate care from their CHPs and peripheral health facilities. We then stipulate that community health promoters with training, adequate supplies and consistent supervision and support, will be able to test for, and treat uncomplicated cases of malaria in the community, and refer cases of malaria in pregnancy, children under 5 or severe malaria to the nearest health facility. Finally, a health system with well trained and equipped health providers will be able to efficiently diagnose, treat and follow-up patients with malaria - reducing new cases as well as providing quality care for those with malaria at both community and facility levels (Fig 2).

Using the conceptual framework we were able to develop in-depth interview guides and focused group discussion guides with the questions covering the main topics of: key roles and responsibilities related to malaria (stakeholder mapping), knowledge and understanding of malaria (cause, transmission, at risk groups), relevant training related to malaria, malaria prevention methods, screening (signs and symptoms), diagnosis and treatment of malaria, supportive supervision (by CHPs, CHAs and sub-county managers), community perceptions on the care that they receive from their CHPs and when they visit the health facilities (see S1 File. Tools used for data collection in CCMm study in Kilifi).

Notably CCMm was implemented in 10 facilities in 5 out of the 7 sub-counties. We purposely selected all the 10 facilities where CCMm was implemented and 4 additional facilities to understand the standard of care for malaria patients where CCMm was not implemented. We aimed to get a variation of facilities located in rural vs urban areas, high vs low workload and malaria endemicity, and run by a mix of male and female in-charges. At sub-county level we contacted the sub-county medical officers of health and then met with and interviewed the sub-county malaria coordinators (5 out of 7). In each sub-county, two facilities were selected, and in each facility, we aimed to interview the facility in-charge, the community health assistant (CHA - CHP community supervisor); have group discussions with CHPs and focus group discussions with community members (see supporting information S1 File. Tools used for data collection in CCMm study in Kilifi). The CHAs organized and selected the participants of the FGDs with instructions to have a mix between male and female CHPs and community members.

Recruitment and data collection were done between 01/03/2025 and 30/06/2025. All county, sub-county and facility in-charge interviews and feedback meetings were conducted in English by EW and GM. FGDs were conducted in Swahili. Information sheets were reviewed by EW, GM and study participants before each interview or FGD. Verbal and written consent was acquired from all respondents prior to all IDIs and FGDs. Key informants signed written consent that was witnessed and co-signed by the interviewers EW or GM. Two members of each FGD signed written consent on behalf of the group. Information sheets were given to all respondents to retain in case they had future questions or complaints. Each participant retained a copy of the information sheet and signed consent form. All IDIs and FGDs were digitally recorded, the transcripts transcribed verbatim, and Swahili FGD transcripts translated to English.

### Ethical considerations

Ethical approval for this study was obtained from Pwani University (ISERC/EXT/001/2025), Kilifi County Department of Health and a research permit from the National Commission for Science Technology & Innovation (NACOSTI/P/25/415619). Prior to participation, written informed consent was obtained from stakeholders at county, sub-county, facility and community level. For community focus group discussions, two participants signed the consent form on behalf of the group. All participants were compensated for their time according to local guidelines.

### Demographic characteristics of participants and summary of data collected

Table 1 below shows a summary description of key characteristics of the key informants interviewed. More than half of the CHPs interviewed were female (62%) and around the age of 35–44 years, with an average of about 10 years’ experience as a CHP. Facility in-charges were mostly male with majority under the age of 44 years and with average experience of about 5 years. Despite an effort from the CHAs to include equal representation of gender, more female community members participated in the focus group discussions (66%). Additionally, the majority of the community members reported having some exposure to formal education, with more than half attaining a primary level of education and only a few receiving tertiary education (8%).

**Table 1:** Demographic characteristics of participants involved in CCMm study.

Demographic characteristic	Category	Number of participants	Percentage or mean
**CHPs (n = 42)**			
Gender	Female	26	62
	Male	16	38
Age (In years)	25-34	7	16.7
	35-44	17	40.5
	45-54	12	28.6
	55-64	6	14.3
Avg. time as CHPs	1-26 years		10.2
Avg. number of households	26-350		67
**In-Charges (n = 14)**			
Gender	Female	4	28.6
	Male	10	71.4
Age (Years)	25-34	5	50
	35-44	4	40
	45-54	1	10
Avg. time as ICs	1-26 years		4.8
**Community members (n = 76)**			
Gender	Female	50	65.8
	Male	26	34.2
Average Age of community members	42 years		Range: 21–77
Level of education	No formal education	7	9.2
	Any primary education	45	59.2
	Any secondary education	17	23.7
	Any tertiary education	6	7.9
Number and use of LLINs (number of LLINs vs number of sleeping spaces in the household)	Number of LLINs = number of sleeping spaces	74	97.4
	Number of LLINs = number of sleeping spaces	2	2.6
	Number of LLINs = number of sleeping spaces	0	0
From community members in the FGDs, how many slept under a net	Yes	74	97.4
	No	2	2.6

A total of 28 in-depth interviews (5 county and sub-county managers, 10 facility in-charges, 12 CHAs and 7 CHPs), 11 group interviews (where CHPs form the same facility were interviewed together in groups of 3), 12 FGDs (at least 6 participants with no prior knowledge of each other) and 3 feedback meetings were conducted during the study period.

### Data analysis

All audio recordings were converted into digital text format for analysis. Audio recordings from in-depth interviews (IDIs) and focus group discussions (FGDs) were transcribed verbatim. Notes taken during stakeholder feedback sessions were typed using Microsoft Word and PowerPoint. Any content recorded in Swahili was translated into English. All textual data were imported into NVivo 11 qualitative data analysis software to facilitate thematic framework analysis, following the approach described by Gale et al.[16].

Preliminary thematic analysis began in the field on 07/03/2025 led by EW during and immediately following the IDIs, FGDs, and stakeholder feedback meetings held at the facility, sub-county and county level. These early analyses helped shape and validate emerging themes and the direction of subsequent data collection. Analysis was structured around four primary domains of inquiry:

1. Stakeholder understanding of malaria and CCMm
2. Activities carried out as part of CCMm training and support
3. Stakeholder experiences of the implementation of CCMm noting:

- Perception of the care that patients receive from their CHPs and at the facilities that they visit specifically related to malaria testing, treatment, health education
- Enabling factors and perceived barriers

4. Contextual factors affecting the implementation of CCMm including:

- Integration with existing reporting structures and health information systems
- Availability of pharmaceutical and non- pharmaceutical supplies (sustainability plans)
- Compensation and working conditions of CHPs

These domains of inquiry guided the construction of the coding tree in NVIVO 11, transcripts were imported and data organized into framework matrices and thematic charts to facilitate comparison and synthesis. Additionally, data interpretation was validated through participatory feedback sessions with various stakeholders including Kilifi County Department of Health managers, frontline health care workers and sub-county managers. Quantitative data was accessed for research purposes and analyzed descriptively from 01/04/2025–31/09/2025 (data extracted was between 01/01/2025 – 30/06/2025). It is reported in proportions, and prevalence reported with 95% CI. Qualitative data were used to triangulate quantitative data and vice versa to validate study findings and ensure comprehensive analysis which was concluded on 31/09/2025.

## Results

### The burden of malaria in the facility catchment areas where CCMm was implemented

Facility data extracted from the KHIS summarizes the positivity rate of malaria across the ten facilities where CCMm was piloted, stratifying it by two age categories, i.e., under 5 years and over 5 years, for the period of 01/01/2025–30/06/2025 (Table 2). Authors did not have access to information that could identify individual participants during or after data collection. The overall positivity rate for malaria in Kilifi County was 21.1% (95% CI: 20.8%–21.4%) during the first half of 2025. This represents a total of 16,196 cases detected using malaria rapid diagnostic tests (RDTs) out of 76, 804 tests reported across Kilifi County. Among the 10 facilities selected for the study, a total of 7,568 malaria cases were reported within the first half of 2025. 1,268 (17%) had been diagnosed and managed by CHPs in the community. The highest positivity rate for malaria among children ≤ 5 years was reported in Lenga dispensary at 94.1% while the lowest positivity rate was reported in Kiwandani dispensary at 4.7%. The highest positivity rate amongst those above ≥ 5 years was reported from Kadzinuni dispensary 73.3% while the lowest was recorded in Kiwandani dispensary 6.7%.

**Table 2:** Health facilities within the 5 sub-counties where CCMm was piloted and the respective malaria positivity rate (01/01/2025 to 30/06/2025) as reported within Kenya Health Information System.

Sub-county	Health facility	Total Tested for malaria < 5 Years	Malaria positive < 5 Years (%)	Total Tested for malaria > 5 Years	Malaria positive > 5 Years (%)
Ganze	Ganze	69	6 (8.7)	361	36 (10)
	Jaribuni	864	118 (13.7)	2651	470 (17.7)
Kaloleni	Kinarani	308	17 (5.5)	859	175 (20.4)
	Mgamboni	227	116 (51.5)	1183	719 (60.8)
Kilifi North	Kadzinuni	879	513 (58.4)	4865	3564 (73.3)
	Kiwandani	86	4 (4.7)	432	29 (6.7)
Kilifi South	Pingilikani	697	272 (39)	2647	1198 (45.3)
	Tunzanani	183	42 (23)	430	163 (37.9)
Rabai	Lenga	17	16 (94.1)	89	64 (71.9)
	Makanzani	41	4 (9.8)	269	42 15.6)

### Contextual changes affecting CCMm

Before CHPs started testing and treating community members with suspected malaria, there was a countywide mass distribution of Long-Lasting Insecticide Treated Nets (LLINs) from October to December 2024, led by the Kenya national malaria control program. Almost all the participants reported receiving and using LLINs. Community members needed their national identification card (ID) and phone number to register to receive LLINs. In some cases, community members who did not have IDs or phone numbers had to be listed under household heads who did. In a few rare cases there were still community members who did not receive the LLINs because they were not in the area during the time of distribution or were erroneously registered in other distant locations due to system errors. CHPs reported that they instructed community members on how to use the LLINs after distribution and remained available for any questions. They reported instructing community members to lay out LLINs in a cool shaded place for 24–72 hours and hang the LLINs using the six points depending on the structure of their sleeping area. Slightly more than half of the community members interviewed reported that they received and understood the instructions on how to prepare and use LLINs. However, there were variations in how they used the LLINs. Some reported using them according to the instructions given by the CHP, while some read the instructions on the LLIN itself. When it came to the maintenance, both CHPs and community members reported that they washed the LLINs with a mild bar soap with water, and the period in between washes depended on the participants. Some CHPs and community members reported washing the LLINs with powdered detergents. Community members reported that when their nets were too worn out with holes, they would sew them. For those who missed nets during mass distribution, they either had to ask CHPs who had extra nets to give them or buy nets from local shops. In addition, pregnant women routinely received IPTp including additional nets at the beginning of their clinic antenatal care (ANC) visits. Subsequently after delivery, they would receive malaria prophylactic medication.

### Community understanding of malaria cause and risk factors

Most community and CHPs interviewed in the CCMm project implementation sites understood malaria to be an illness transmitted by a mosquito. The level of understanding varied among stakeholder groups. Most community members reported that it was an illness caused by “viini” which would translate to small organism or germ, carried by mosquitoes. There were a few who reported that these small organisms could also be found in stagnant water - a misconception corrected by fellow participants during FGDs. Community health workers (CHPs and CHAs) further described malaria as an illness caused by parasites that can be transmitted by a female anopheles’ mosquito, clinical health workers were able to name the different types of parasites (plasmodium falciparum and plasmodium vivax) and their life cycle.

Most respondents in the CCMm project implementation sites reported: not sleeping under a net, living near breeding areas as high-risk factors for getting malaria; with pregnant women, infants, and the elderly getting more severe symptoms. Environmental and hygienic practices including clearing bushes, draining stagnated water and larvicidal spraying of water bodies were correctly reported by CHPs and community members interviewed, as some of the mechanisms for malaria prevention and control. Some community members also mentioned applying mosquito repellents and wearing clothes that covered their hands and feet especially in the evenings. A few community members reported that the smoke from burning the barks of certain trees acted as a mosquito repellent.

In contrast, CHPs in the non-CCMm implementation sites’ knowledge of malaria was limited to risk factors, prevention and identifying signs and symptoms. They reported that they referred all suspected cases to the nearest facility and could not distinguish between complicated and uncomplicated malaria.

### Stakeholder views and experiences on the CCMm training

Most CHPs involved in the implementation of CCMm reported positive views about the conduct of the CCMm training. The reasons provided for the perceived success in training was the fact that they had theoretical training followed by practical training on testing for malaria during the training and then slightly over one month of practical exposure at the facility where CHPs were testing (using RDTs) and treating patients with confirmed uncomplicated malaria. Most CHPs also recommended additional refresher training, especially on testing procedures.

*CHP 2: I really appreciated when at the start we had the training and then practiced pricking on ourselves before we were expected to prick other CHPs and then practice at the facility under the supervision of our in-charge or lab technician, it gave me confidence that I was doing it right when I had to do it on my own out in the village.*

*CHP 3: yes I was really afraid of the blood at first, but I got used to it, the training people were also very friendly and answered all our questions and allowed us to practice until we got all the steps right.*

*CHP 2: there were situations that we were going through with them to cover how to educate the patient at every step, how to manage with the equipment and document everything, and with different types of patients, what to do if the test was not valid or in cases where we had to refer patients.* CHP group interview, Facility 7, 2 female, 1 male

Sub-county malaria coordinators felt that part of the successful training was attributed to the productive working relationships between the county department of health and other CCMm pilot project implementing partners.

*We (sub-county managers) were involved from the beginning and even helped with the technical aspects of the training. Personally, it also helps me to know what to look for when we go for supportive supervision sometimes. I have not heard any complaints from the community.* Sub-county malaria coordinator, sub-county 4, Female

### CHP-Patient CCMm journey map and experiences

**Fig 3:**
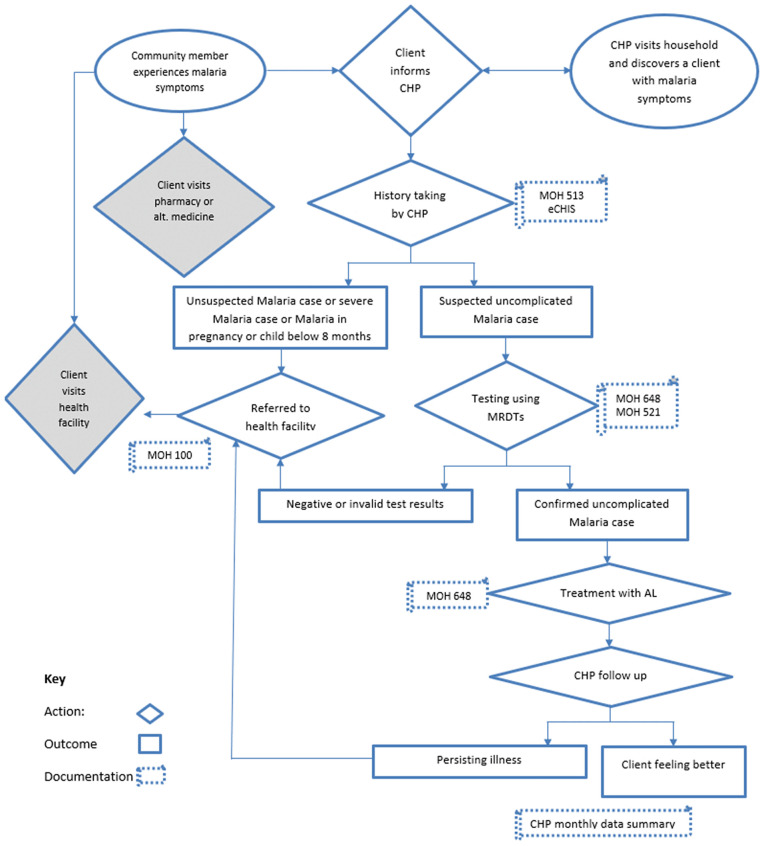
Community health promoter (CHP) - Patient journey map for community case management of malaria in Kilifi.

Community members and CHPs in the CCMm project implementation sites reported that patients with malaria symptoms could be identified by the community members contacting the CHP or be discovered during routine CHP home visits. The CHP would complete household registration and collect demographic data using the CHP community health information system register (MOH 513), and the electronic community health information system (eCHIS). Malaria related history taking including presence of fever and other symptoms would be recorded in the CHP Treatment and Tracking Register (MOH 521). The weight of the patient would also be measured (Fig 3).

Community members and CHPs (who had undergone the CCMm training) had no difficulties in identifying the signs and symptoms of malaria. All CHPs interviewed in the CCMm project implementation sites reported being confident in testing for malaria using RDTs. They reported that once they had identified a community member with suspected malaria, they would explain why they needed to test to be sure that it was malaria before offering treatment. Pregnant women, children under 8 months and patients with symptoms of severe malaria were not tested at home and immediately referred to the facility. CHPs also reported that they described how they were going to do the actual test to the patient. After testing and reading the results, CHPs reported that they relayed the information to their patients. Those with negative test results were referred to the facility for further tests. Those with positive results were offered treatment by the CHP. It is important to note that some community members sort care directly from the health facilities or pharmacies (Fig 3).

CHPs in the control facilities reported that they refer all patients with fever to their nearest facilities. They had heard of CHPs that were able to test and treat malaria in the community, but they did not have in-depth knowledge or confidence to do the same, and they only had thermometers and pain medication in their CHP kits, no RDTs or artemether‐lumefantrine combination treatment.

### Stakeholder experiences, views and perceptions of CCMm

#### Community members’ experiences and views on CCMm.

Community members reported being confident in the abilities of their CHPs to provide general community health services. They had overwhelming appreciation for the new household malaria management skills of their CHPs in the CCMm project implementation sites. They viewed it as something that was positive and should be supported to continue.

*“P7: We really appreciate our CHPs and we trust them to do what is best, we are also very grateful that now we can get the tests (for malaria) done and be attended to at home, especially for those of us who live far away from the hospital, I wish the same could be done for people suffering with blood pressure (Hypertension) and the sugar disease (Diabetes).* Community member FGD, Facility 3

There were no differences reported in the way that CHPs conducted the testing and treatment for malaria in the households. Community members reported that the CHPs always explained what was being done and that they felt free to ask questions at any point.

On facility services, community members reported that they are always well received and appreciated confirmatory laboratory tests. The community members in two facilities complained about the poor attitude and lateness of their facility in-charge.

*“P5: Most of the days they (facility staff) come to the facility at 10 or 11, and we have been waiting since 8 in the morning, when you ask or complain, they are very harsh, and they* *go for a long lunch break and close by 3pm; but that is even better, sometimes there is no one in the facility at all”* Community member FGD, Facility 2

Community members also reported that all pregnant women were given LLINs during their first ANC visit and after delivery, but not all pregnant women got prophylactic malaria treatment during their ANC visits. Almost all the community members complained about the waiting times at the facility, regardless of whether they were referred by a CHP or not. Quite a number of facilities did not have staff quarters near the facility and facility staff had to commute from great distances using unpredictable public transport. Most facilities were also closed at night and during weekends.

Other challenges mentioned by community members include times when CHPs run out of test kits and medication and they must go to the facility or buy medication because even the facility has no medicines available.

#### CHP experiences and perceptions of CCMm.

All CHPs in the CCMm project implementation sites reported positive opinions on the need and usefulness of CCMm. Those trained in CCMm expressed a renewed confidence in their practice. Most CHPs had received the new CHP field kits and were eager to add RDTs and artemether/ lumefantrine to them and offer CCMm services to their communities. CHPs in the CCMm project implementation sites also reported that community response and acceptance of their new roles was positive due to the established trust with community members, a combination of supportive supervision from their CHAs and supervisors, and increased facility and legitimacy from their interactions with the county department of health and implementation partners like *Amici del Mondo-World Friends ETS Kilifi*, should continually be promoted to keep up CHP motivation and commitment to their roles.

*Before we started testing and treating patients in their homes, there was a meeting that was called and we had it in our village where the community health assistant and some of the facility staff let the community know that we CHPs would now test and treat those with malaria at home. They received us well and now, sometimes they even come to our homes or call us if someone is not feeling well and we go and see them.* Community health promoter 2, Facility 7

### Household treatment of confirmed uncomplicated malaria by CHPs

In the CCMm project implementation sites, CHPs reported that all cases with a positive RDT test were treated. Treatment was based on age and weight brackets as described in national guidelines and training materials (S2 File. Treatment schedules and list of documents - extracts from CHP training guidelines).

CHPs also reported that the first dose was given at home under their supervision to observe if the patient would tolerate or vomit the administered artemether and lumefantrine treatment. They then explained how to take the rest of the medication and answered questions, mostly on the timing and number of tablets to take. Three days after the initial treatment, CHPs would follow up on their patients to make sure that they completed taking their medication and to check on the condition of their patients. Community members did not report any difficulties in following the instructions on how to take their medication.

In the non-CCMm project implementation sites, CHPs in the control facilities reported that they only gave patients paracetamol and referred them to their nearest facility for treatment. In one facility, the in-charge had trained the CHPs to test and treat patients at the facility, and they were able to. It was unclear whether this practice was known or authorized at county level.

There were no reported differences in how testing was conducted (based on age, gender, length of time one had served as a CHP, or the number of households that a CHP oversaw). Although there were no differences in how the procedure was conducted, there were some deviations in the time they had to wait on the results.

*P3: The amount of time that you have to wait depends on the buffer that you are using, some say wait for 15 minutes, some say 20 minutes … it is written on the packet. Some will even start to see the results after 5 minutes* Community health promoter 3, Facility 9

CHPs reported no difficulties in reading and explaining the test results to their patients. One line on the cassette indicating a negative result and two lines indicating positive results. A few reported cases of invalid test results requiring them to repeat the test. Two CHPs in two different facilities reported that they had cases that were negative from RDTs but turned positive for malaria when their patients visited the health facility and were tested using microscopy.

The CHPs on the other hand reported the need for refresher training on the testing procedures. During one of the supervision sessions with *Amici del Mondo-World Friends ETS* (CCMm implementation support partners in Kilifi) team, it was reported that CHPs had a challenge in waste disposal especially regarding the prickers.

### Challenges faced by CHPs in implementing CCMm

CHP experiences on challenges in implementing CCMm were reported as: inefficient referral systems (CHPs did not always get feedback regarding the patients they referred to the health facilities); inadequate and inconsistent compensation from the county and national government; out of pocket expenses like transport and airtime (essential when CHPS could not afford transport to follow-up on patients in their homes), and a lack of equipment (bags, umbrella, boots, caps, phones/tablets, torches, motorbikes, bicycles, raincoats). CHPs also reported inconsistent and inadequate supplies of artemether‐lumefantrine treatment in general. Stock outs for artemether‐lumefantrine were more frequently reported as a challenge, more than stock outs for RDTs which were mostly limited to specific bands (dosage categories).

*“Sometimes we do not have all the available bands, especially band 6 (weight category 5 to 15 Kgs). In those cases, we advise CHPs to refer the patient to the facility so that we can reorganize other bands to provide the correct dose for them”* Community health promoter 1, Facility 6

They also reported difficulties in maintaining both digital (eCHIS) and paper records which had to be reviewed and corrected in meetings with the community health assistants before being sent to the sub-county. Following up on referrals and tracking referral forms was a challenge that remained unresolved in most facilities.

“*There are really many documents to fill … for every patient you start with the household register, if you are referring them, you write them a referral form, which you don’t know if you will get back from the patient. If they have malaria, then you also must record different documents to track the number of people you have tested and treated and how you have used RDTs and any medication given. On top of that you must put in that information on eCHIS, your monthly report and the World Friend’s digital form. Our CHA (community health assistant) usually goes through our monthly reports before submitting them to the sub-county, the digital records are harder to keep updated.”* Community health promoter 1, Facility 4

These persisting challenges if not promptly addressed present significant obstacles to the implementation of CCMm and other community-based health programs.

### Health worker experiences, views and perceptions of CCMm

Health facility staff were highly appreciative of CCMm and the work that CHPs had done, noting that they had significantly reduced the workload at facility level and in some cases even reduced the number of re-visits as the follow-up was done in the community by the CHP. They reported that clearer procedures and reporting channels need to be reinforced to be able to represent the roles of CHPs, reduce duplication data of malaria testing and treatment data, as well as strengthen referral and patient/CHP feedback channels.

Nonetheless, facility in-charges reported challenges in the number of available staff including lab technicians with some facilities training non-laboratory staff to carry out malaria tests. Of the ten implementation facilities, only three facilities had staff accommodation (two functional), and most facility staff had to commute great distances to work. They also emphasized supply chain inefficiencies in the distribution of RDTs, artemether-lumefantrine and intravenous artesunate. One facility reported that they had to organize to borrow supplies from another facility that had a lower incidence of Malaria cases with the authority from their sub-county malaria coordinator

County and sub-county health managers reported that CHPs were carrying out their roles according to the training they gave them and in accordance with malaria prevention and management guidelines. Health managers at sub-county level felt confident in their capacity to identify malaria hot spots and high malaria seasons and respond accordingly. They also mentioned that the CCMm has improved the quality of the data they receive at sub-county level.

*“Initially, before CCMm, CHP had difficulties in understanding and documenting malaria indicators and we used to get big gaps in the data submitted but now through the support supervision and mentorship of us and their supervisors, at least there are improvements, and currently, even we can attest we only get a few errors in the details but overall, there is harmony in all the tools and better data”* sub-county malaria coordinator 3

A summary of stakeholder perceptions on CCMm and the factors affecting its implementation is shown in table 3 below.

**Table 3:** Stakeholder perceptions on the implementation of CCMm.

Stakeholder	Currently working	Perceived challenges	Determinants of success
Community	• CHPs offering testing and treatment of malaria in the community • Community trust and acceptance has been maintained • Cost of facility visits for malaria has been eliminated • Perceived appropriate use of ITNs	• Access: ITNs, Facility, Medication, • Referral • Difficult interactions with facility staff • SHA	• Community trust mind-set promotes acceptability of CHP new roles • Dialogue • Social behavior change
CHP	• Able to offer CCM • Community trust and acceptance • Supportive supervision	• Inadequate and inconsistent compensation • Transport • Equipment • Referral completeness	• Appreciation; financial and non-financial • Training esp. OJT • Confidence • Supportive supervision
CHA	• CCM • Good relationships with CHPs and ICs • Reporting	• Transport • Dealing with different age groups	• Power dynamics • Supportive supervision with compiling CHP data
Facility	• Reduced workload of patients with malaria • Treatment of severe cases of malaria • IPTp – SP • Supportive supervision	• Inadequate diagnostic abilities • Push system of allocation • Stock outs • Staff shortages	• Availability of resources including staff, medication and RDTs • A functional and staffed lab
Sub-county	• CCM • Reporting	• Resources • Sustainability plans	• Integration of CCMm into existing referral and health information systems • Leadership skills -emersion

### Other factors affecting implementation of CCMm

Other contextual factors reported to affect the implementation of CCMm include: the integration of data generated from CCMm with existing reporting structures and health information systems; the availability of pharmaceutical and non- pharmaceutical supplies long term; as well as sustainability plans after the exit of implementation partners.

## Discussion

Community Case Management (CCMm) continues to be an essential strategy in the global fight against malaria, particularly in sub-Saharan Africa where access to health facilities remains a challenge for large segments of the population [17]. Its importance in malaria control stems from its ability to extend lifesaving services to remote and underserved populations, using community health workers [10] to ensure prompt diagnosis and treatment—key components of effective malaria management. Kenya’s journey in community-case management of malaria reflects a progressive model. Its foundation is based on the progression from the community health strategy [18] that introduced community health workers who are now compensated community health promoters. The utilization of CHPs in improving access to malaria treatment concurrently evolved with Kenya's test and treat policy [19]which emphasized facility testing for malaria before treatment; as well as integrated community case management [20]which stated that when community health workers are trained, supervised and supported, they are able to have adequate knowledge, skills and competency to provide quality services including treatment of sick children. CCMm in Kenya has since been implemented in different counties in Kenya.

The overall positivity rate for malaria in Kilifi County was 21.1% (95% CI: 20.8%–21.4%). This high positivity rate demonstrates that Kilifi has an ongoing malaria epidemic, and that more efforts are required in both prevention and early detection of these cases. The (Kenya Malaria Indicator Survey) KMS 2019–2023 classifies Kilifi as endemic for malaria transmission [9]. Other parts of the country have also reported high prevalence of malaria, for example, 13.7% in Nandi County in 2024 [21]. This agrees with the World Malaria Report 2024 which reported that malaria prevalence in Southeast Asian countries has been shrinking significantly, while tropical Africa continues to experience high malaria prevalence [22]. Within the 10 health facilities where CCMm was being piloted, there were 7,568 confirmed malaria cases, of which 1,268 (17%) were diagnosed and treated by CHPs, showing the impact that CHPs can have on increasing access to diagnostics and treatment for the members of the community, while also reducing malaria related workload at facilities. Efforts should be made to increase the number of CHPs, especially among the facilities that reported high numbers of positive malaria cases at the facilities, while also trying to understand why some patients still opted to go to the facility for malaria services.

The positivity rate for malaria both among the children below 5 years and among individuals above 5 years varied widely across the 10 facilities where CCMm was being piloted, suggesting that we have malaria hotspots within Kilifi County. Bejon. P *et al*, reported observing hotspots within hotspots, down to the level of individual homesteads within Kilifi and our finding re-enforce this observation. These malaria hotspots ought to be mapped, and a more customized CCM approach could be implemented within these areas. While hotspots can undermine control strategies, targeting interventions at transmission clusters or ‘hotspots’ may prove very effective [23]; this approach might also benefit CCMm.

Our study findings show that the implementation of CCMm is reliant on different community, CHP, and health system related factors. Community members are accepting of and benefiting from CHPs who offer testing and treatment services for malaria in households. This has helped maintain trust, eliminate costs associated with facility visits and promote the use of LLINs. They express that the success of CCMm is still dependent on the availability of test kits and medications to CHPs as well as at the facility level. Stakeholders in the implementation areas are confident that CHPs are effectively delivering CCMm with acceptance from the community and supportive supervision from their peers and supervisors. Despite this, CHPs struggle with logistical issues (lack of transport to visit far off households); inadequate and inconsistent compensation; periodic stock outs of RDTs and artemether-lumefantrine medication; and issues with broken feedback channels especially in completing and following up on referrals. Community health assistants (CHAs) are actively involved in CCMm through monthly supervision meetings with CHPs and facility staff contributing to improved reporting on malaria indicators. Health facility staff are also benefiting from a reduced burden of malaria cases so that they can focus on IPTp and the treatment of severe malaria cases. Nonetheless, facilities face significant challenges in diagnostic capacity, supply chain inefficiencies (for RDTs, artemether-lumefantrine and intravenous artesunate), staff shortages and difficulties in accessing rural facilities.

Community members in our study were all confident in their CHPs’ abilities to deliver community health services. They specifically appreciated the malaria testing and treatment services that their CHPs are delivering in the household. As mentioned earlier, this could be attributed to consistent engagement and trust with their CHPs making them more likely to accept new services. This is unique to this intervention where communities were involved and participated in the design of the intervention, there was full support from the county and local administration in terms of community sensitization and education, and mass distribution of LLINs and training of CHPs happened before the start of CCMm. This ensured that communities were aware of the services available to them and had adequate support to make good health decisions in terms of malaria prevention and health seeking behavior. Continuous health education about malaria has also led to less and cautious use of alternative/traditional medicines. Sustained and effective social behavior change (SBC) interventions are vital for achieving optimal uptake of CCMm. Other similar programs struggled with community acceptance because initial community sensitization was often weak; and CHWs lacked support in raising awareness [24].

Community health promoters (CHPs), the equivalent of community health workers, are essential to the implementation of CCMm. Our study shows high community acceptance and appreciation of CHPs and the services they provide including CCMm. These findings are augmented by similar studies in Kenya and SSA [25] and contrast other findings challenging the fidelity [26] of using CHWs in providing community-based testing and treatment for malaria [27]. Similar studies confirm that the success of CHP community-based programmes require that CHWs have enough training and practice time, and standardized implementation to encourage good performance at scale [28,29]. The use of RDTs by community health workers (CHWs) in diagnosis has been proven to reduce overuse of antimalarials [30] as it reduces prescribing of antimalarials to people without malaria [29]. CCMm also reduces the burden on overstretched health facilities by managing uncomplicated cases at the community level. This decentralized care can improve the health system cost-effectiveness [31] by reducing transportation costs for patients, improving treatment-seeking behaviour, and preventing the progression to complicated malaria which is more expensive to treat. While CCMm has proven to be an effective strategy to increase access to prompt and appropriate treatment, especially in remote or underserved areas, its success hinges on the program design and the enabling environment in which it is deployed.

Another important aspect in the implementation of CCMm is the diligence of CHPs in following up on patients after treatment to enhance adherence to treatment and safety [32]. Our findings show that while this is desirable, CHPs experienced challenges in following up on their malaria patients due to logistical issues including lack of transport to travel back to their home. To counter this, they used the limited airtime provided by implementing partners to follow up most patients who received treatment from them with a call. The greater challenge was following up patients who had been referred to facilities for further testing or because they had complicated malaria. This is due to broken referral systems which resulted in inconsistent filling in of the referral forms by the facility health workers back to the CHP. This made it extremely difficult for CHPs to know whether patients went to the facility and whether they received appropriate healthcare services. Studies in similar contexts recommend policies that ensure supportive relationships exist between primary health care centres and CHPs, as well as training clinical staff on the importance of documenting outcomes of cases referred from the community [33].

CHPs in our study also mentioned difficulty in reporting both on paper and electronically. These challenges were addressed by ensuring they had monthly supervision from their supervisors (community health assistants) to correct any misrepresentation or missing data at community level. There is however a lack of integration with the current health management information system, there is no way of currently evaluating the accuracy of data entered at community level with HIMIS or eCHIS, even for our study we could only access aggregated data and therefore could not assess the performance of individual CHPs when it came to malaria reporting. CHPs also expressed dissatisfaction with their compensation [34] and recognition for their work, findings that remain unchanged in Kenya and other countries with CHWs [24].

Implementation of CCMm in Kenya and more widely faces significant health system challenges [24]. CCM is specifically more relevant in a context with constrained resources because it acknowledges the key constraints in access to health facility services and helps ease the high facility health worker workload by shifting tasks like testing and treating uncomplicated malaria from high volume health facilities to CHPs in community health units, making the health system more resilient. Our study findings corroborate these benefits in the expansion of access to malaria testing and treatment, as well as added benefits in improving CHP competency and community trust. Evidence from other Kenyan counties also shows that CHPs have been able to deliver effective care for uncomplicated malaria and improve efficiency of referral for clients with severe cases of malaria using already existing human resources (CHPs), with additional training [12]. In addition to trained health workers, it is also paramount to have a consistent and adequate supply chain of RDTs and artemether‐lumefantrine at community level as well as microscopy and medication to treat complicated malaria at facility level. CHPs and facility in-charges in our study reported inadequate supplies of specific batches of AL, while RDTs were provided by the implementation partner. Supportive supervision, while of good quality, happens less frequently due to transport costs and availability of teams. A clear sustainability plan for the county to expand the reach of CCMm and provide the needed RDTs and continue to supply antimalarials will be critical before the end of the intervention. There is a pressing need to integrate CCMm into domestic public financing with budget lines for CHW compensation, logistics, and supervision. Without predictable financial support, CCMm risks losing effectiveness and cannot scale nationwide.

### Recommendations and conclusion

If the county was considering expansion, the program design would have to embed internal, monitoring and evaluation systems to ensure implementation fidelity and enable evidence-based decision-making. Supportive supervision needs to incorporate both data driven and competency-based evaluations. This is also in a context with the already challenging integration of community level data into HMIS. Digital tools effectively used by CHPs could provide relief to the double entry requirements and make integration with national and facility level health information systems more efficient. Streamlined reporting mechanisms and robust monitoring and evaluation (M&E) systems are essential to guide implementation and scale-up. Additionally, referral systems with accountability feedback loops between communities and higher levels of the health system should be developed and strengthened to provide universal coverage. The increasing use of AI in health is also a timely consideration to consider ways in which electronic health records, telehealth platforms, and remote patient monitoring can be leveraged to improve data sharing and management [35].

Health managers in our study emphasized the need for strong collaboration among the Ministry of Health, County Health Teams, NGOs, and community-based organizations - clear governance frameworks are essential to streamline roles, ensure accountability, and align county and national malaria strategies. Countries that integrate CCM into national malaria control strategies and provide supportive legislation tend to have more success with implementation [36]. While malaria CCM is often introduced as a vertical intervention, integrating it with broader primary healthcare services, including iCCM can enhance efficiency and sustainability. However, integration can also pose challenges in terms of increasing the workload for CHPs and complicating supply chains [37,38].

Malaria transmission patterns in Kenya are influenced by climatic and environmental factors, CCMm programs must be flexible enough to adapt dynamically to seasonal transmission fluctuations by increasing CHW readiness, stock levels, and community messaging ahead of peak prevalence periods [39,40].

Stakeholder experiences from our study highlight both the potential benefits and the practical constraints of implementing CCMm in Kilifi County. It is important to note that the county department of health and implementing partners deliberately created and maintained an enabling environment with effective training, supportive supervision, and reliable supply of RDTs and antimalarials. This may not continue when the program comes to an end, but the foundation laid in terms of skills transferred to CHPs and lessons learnt from the implementation process remain useful, especially for scale up. Addressing systemic and resource-related barriers is essential to maximize the equity and effectiveness of malaria interventions at the community level. Interventions should be designed with the involvement of communities as sensitization and equity-focused communication will increase acceptance and utilization across all socioeconomic groups. Institutionalization of CHW incentives and career pathways, together with refresher training and continued supportive supervision for both CHPs and health workers, and emphasizing diagnostics, referral systems, data reporting and good governance will improve CHP retention and motivation; and provide evidence for effective, evidence-based decision making. These findings can inform policy and program adjustments for strengthening community-based malaria control efforts in Kenya and similar contexts.

## Limitations of the study

This piloted model of the CCMm is not easily replicable: the main partner (WF) provided ongoing technical and resource support, which may not be feasible in other contexts, creating a risk of overclaiming generalizability. The malaria data available from KHIS had missing data in some sections and we are not certain of the accuracy of the data. The outcome indicators are not strongly structured; the focus is more on implementation than on measurable results. Data collection did not include a costing analysis and therefore benefits focus on the number of clients who got tested and confirmed cases of malaria, time-driven activity-based costing may provide more detailed indication of time and cost saved through CCMm. Impacts on malaria prevalence cannot therefore be solely attributable to the implementation of CCMm as other players continue to equally put their efforts on matters prevention of malaria. The use of aggregated data limits the evaluation of individual-level impact reducing accuracy. Despite the limitations the lessons learned during the pilot of CCMm will inform expansion plans across the County and the region at large.

## Supporting information

S1 File Tools used for data collection in CCMm study in Kilifi. (DOCX)

S2 File Treatment schedules and list of documents - extracts from CHP training guidelines. (DOCX)

## References

[1] World malaria report 2025: addressing the threat of antimalarial drug resistance. Geneva: World Health Organization. 2025.

[2] Cohen JM, Smith DL, Cotter C, Ward A, Yamey G, Sabot OJ, et al. Malaria resurgence: a systematic review and assessment of its causes. Malar J. 2012;11:122. doi: 10.1186/1475-2875-11-122 22531245 PMC3458906

[3] Sikaala CH, Dlamini B, Lungu A, Fakudze P, Chisenga M, Siame CL, et al. Malaria elimination and the need for intensive inter-country cooperation. a critical evaluation of regional technical co-operation in Southern Africa. Malar J. 2024;23(1):62. doi: 10.1186/s12936-024-04891-5 38419105 PMC10903059

[4] WHO. Global technical strategy for malaria 2016-2030. 2015. Contract No.: ISBN 978 92 4 1564991.

[5] Hemingway J, Ranson H, Magill A, Kolaczinski J, Fornadel C, Gimnig J, et al. Averting a malaria disaster: will insecticide resistance derail malaria control?. Lancet. 2016;387(10029):1785–8. doi: 10.1016/S0140-6736(15)00417-1 26880124 PMC6215693

[6] Weiss DJ, Bhatt S, Mappin B, Van Boeckel TP, Smith DL, Hay SI, et al. Air temperature suitability for Plasmodium falciparum malaria transmission in Africa 2000-2012: a high-resolution spatiotemporal prediction. Malar J. 2014;13:171. doi: 10.1186/1475-2875-13-171 24886586 PMC4022538

[7] Ashley EA, Dhorda M, Fairhurst RM, Amaratunga C, Lim P, Suon S, et al. Spread of artemisinin resistance in Plasmodium falciparum malaria. N Engl J Med. 2014;371(5):411–23. doi: 10.1056/NEJMoa1314981 25075834 PMC4143591

[8] Elnour Z, Grethe H, Siddig K, Munga S. Malaria control and elimination in Kenya: economy-wide benefits and regional disparities. Malar J. 2023;22(1):117. doi: 10.1186/s12936-023-04505-6 37029370 PMC10080938

[9] Ministry of Health Division of National Malaria Programme K. Kenya Malaria Indicator Survey 2020. Nairobi: Ministry of Health Division of National Malaria Programme. 2023.

[10] Smith Paintain L, Willey B, Kedenge S, Sharkey A, Kim J, Buj V, et al. Community health workers and stand-alone or integrated case management of malaria: a systematic literature review. Am J Trop Med Hyg. 2014;91(3):461–70. doi: 10.4269/ajtmh.14-0094 24957538 PMC4155545

[11] Ratovoson R, Garchitorena A, Kassie D, Ravelonarivo JA, Andrianaranjaka V, Razanatsiorimalala S, et al. Proactive community case management decreased malaria prevalence in rural Madagascar: results from a cluster randomized trial. BMC Med. 2022;20(1):322. doi: 10.1186/s12916-022-02530-x 36192774 PMC9531497

[12] Otambo WO, Ochwedo KO, Omondi CJ, Lee M-C, Wang C, Atieli H, et al. Community case management of malaria in Western Kenya: performance of community health volunteers in active malaria case surveillance. Malar J. 2023;22(1):83. doi: 10.1186/s12936-023-04523-4 36890544 PMC9993668

[13] Sirima SB, Konaté A, Tiono AB, Convelbo N, Cousens S, Pagnoni F. Early treatment of childhood fevers with pre-packaged antimalarial drugs in the home reduces severe malaria morbidity in Burkina Faso. Trop Med Int Health. 2003;8(2):133–9. doi: 10.1046/j.1365-3156.2003.00997.x 12581438

[14] Programme M. Kenya malaria strategy 2023-2027.

[15] Fisher WA, Harman J. The Information-Motivation-Behavioral Skills Model: A General Social Psychological Approach to Understanding and Promoting Health Behavior. 2003.

[16] Gale NK, Heath G, Cameron E, Rashid S, Redwood S. Using the framework method for the analysis of qualitative data in multi-disciplinary health research. BMC Med Res Methodol. 2013;13:117. doi: 10.1186/1471-2288-13-117 24047204 PMC3848812

[17] Christopher JB, Le May A, Lewin S, Ross DA. Thirty years after Alma-Ata: a systematic review of the impact of community health workers delivering curative interventions against malaria, pneumonia and diarrhoea on child mortality and morbidity in sub-Saharan Africa. Hum Resour Health. 2011;9:27. doi: 10.1186/1478-4491-9-27 22024435 PMC3214180

[18] Ministry of Health NK. Kenya Community Health Strategy 2020-2025. 2020.

[19] Amboko B, Stepniewska K, Malla L, Machini B, Bejon P, Snow RW, et al. Determinants of improvement trends in health workers’ compliance with outpatient malaria case-management guidelines at health facilities with available “test and treat” commodities in Kenya. PLoS One. 2021;16(11):e0259020. doi: 10.1371/journal.pone.0259020 34739519 PMC8570506

[20] Onono M, Abdi M, Opondo I, Okung’u J, Asadhi E, Nyamai R, et al. Using the RE-AIM framework to evaluate the implementation of integrated community case management in Kenya. Acta Paediatr. 2018;107 Suppl 471:53–62. doi: 10.1111/apa.14662 30570791

[21] Githinji GK, Odhiambo FO, Andala CM, Chepkwony D, Sang JK, Owiny M, et al. Role of surveillance data in detecting malaria outbreaks in an epidemic-prone region in Kenya: findings from an investigation of a suspected outbreak in Nandi County. Malar J. 2024;23(1):372. doi: 10.1186/s12936-024-05216-2 39695721 PMC11658296

[22] WHO. World malaria report 2024. 2024.

[23] Bejon P, Williams TN, Nyundo C, Hay SI, Benz D, Gething PW, et al. A micro-epidemiological analysis of febrile malaria in Coastal Kenya showing hotspots within hotspots. Elife. 2014;3:e02130. doi: 10.7554/eLife.02130 24843017 PMC3999589

[24] Boakye MDS, Owek CJ, Oluoch E, Wachira J, Afrane YA. Challenges of achieving sustainable community health services for community case management of malaria. BMC Public Health. 2018;18(1):1150. doi: 10.1186/s12889-018-6040-2 30285684 PMC6167894

[25] Marita E, Langat B, Kinyari T, Igunza P, Apat D, Kimori J, et al. Implementation of community case management of malaria in malaria endemic counties of western Kenya: are community health volunteers up to the task in diagnosing malaria?. Malar J. 2022;21(1):73. doi: 10.1186/s12936-022-04094-w 35248055 PMC8897909

[26] Chipukuma HM, Halwiindi H, Zulu JM, Azizi SC, Jacobs C. Evaluating fidelity of community health worker roles in malaria prevention and control programs in Livingstone District, Zambia-A bottleneck analysis. BMC Health Serv Res. 2020;20(1):612. doi: 10.1186/s12913-020-05458-1 32615960 PMC7331272

[27] Druetz T, Ridde V, Kouanda S, Ly A, Diabaté S, Haddad S. Utilization of community health workers for malaria treatment: results from a three-year panel study in the districts of Kaya and Zorgho, Burkina Faso. Malar J. 2015;14:71. doi: 10.1186/s12936-015-0591-9 25889306 PMC4329655

[28] Tayler-Smith K, Kociejowski A, de Lamotte N, Gerard S, Ponsar F, Philips M, et al. Free treatment, rapid malaria diagnostic tests and malaria village workers can hasten progress toward achieving the malaria related millennium development goals: the Médecins Sans Frontières experience from Chad, Sierra-Leone and Mali. J Public Health Afr. 2011;2(1):e12. doi: 10.4081/jphia.2011.e12 28299053 PMC5345471

[29] Ruizendaal E, Dierickx S, Peeters Grietens K, Schallig HDFH, Pagnoni F, Mens PF. Success or failure of critical steps in community case management of malaria with rapid diagnostic tests: a systematic review. Malar J. 2014;13:229. doi: 10.1186/1475-2875-13-229 24924295 PMC4084582

[30] Allen EN, Wiyeh AB, McCaul M. Adding rapid diagnostic tests to community-based programmes for treating malaria. Cochrane Database Syst Rev. 2022;9(9):CD009527. doi: 10.1002/14651858.CD009527.pub3 36073718 PMC9453882

[31] Daviaud E, Besada D, Leon N, Rohde S, Sanders D, Oliphant N, et al. Costs of implementing integrated community case management (iCCM) in six African countries: implications for sustainability. J Glob Health. 2017;7(1):010403. doi: 10.7189/jogh.07.010403 28702174 PMC5502705

[32] Cassidy-Seyoum SA, Chanpheakdey P, Meershoek A, Hsiang MS, von Seidlein L, Adhikari B, et al. Community patient follow-up as a part of P. vivax case management in Cambodia: a mixed methods study. BMC Health Services Research. 2025.

[33] Asiedu A, Haws RA, Gyasi A, Boateng P, Malm K, Ntumy R, et al. Improving Malaria Case Management and Referral Relationships at the Primary Care Level in Ghana: Evaluation of a Quality Assurance Internship. Glob Health Sci Pract. 2023;11(6):e2300050. doi: 10.9745/GHSP-D-23-00050 38135513 PMC10749655

[34] Musoke D, Nyashanu M, Bugembe H, Lubega GB, O’Donovan J, Halage AA, et al. Contested notions of challenges affecting Community Health Workers in low- and middle-income countries informed by the Silences Framework. Hum Resour Health. 2022;20(1):4. doi: 10.1186/s12960-021-00701-0 34991590 PMC8734299

[35] Awosiku OV, Gbemisola IN, Oyediran OT, Egbewande OM, Lami JH, Afolabi D, et al. Role of digital health technologies in improving health financing and universal health coverage in Sub-Saharan Africa: a comprehensive narrative review. Front Digit Health. 2025;7:1391500. doi: 10.3389/fdgth.2025.1391500 40453811 PMC12122447

[36] Strachan C, Wharton-Smith A, Sinyangwe C, Mubiru D, Ssekitooleko J, Meier J, et al. Integrated community case management of malaria, pneumonia and diarrhoea across three African countries: A qualitative study exploring lessons learnt and implications for further scale up. J Glob Health. 2014;4(2):020404. doi: 10.7189/jogh.04.020404 25520794 PMC4267083

[37] Sunguya BF, Mlunde LB, Ayer R, Jimba M. Towards eliminating malaria in high endemic countries: the roles of community health workers and related cadres and their challenges in integrated community case management for malaria: a systematic review. Malar J. 2017;16(1):10. doi: 10.1186/s12936-016-1667-x 28049486 PMC5209914

[38] Allen KC, Whitfield K, Rabinovich R, Sadruddin S. The role of governance in implementing sustainable global health interventions: review of health system integration for integrated community case management (iCCM) of childhood illnesses. BMJ Glob Health. 2021;6(3):e003257. doi: 10.1136/bmjgh-2020-003257 33789866 PMC8016094

[39] Le PVV, Kumar P, Ruiz MO, Mbogo C, Muturi EJ. Predicting the direct and indirect impacts of climate change on malaria in coastal Kenya. PLoS One. 2019;14(2):e0211258. doi: 10.1371/journal.pone.0211258 30726279 PMC6364917

[40] Ryan SJ, Lippi CA, Zermoglio F. Shifting transmission risk for malaria in Africa with climate change: a framework for planning and intervention. Malar J. 2020;19(1):170. doi: 10.1186/s12936-020-03224-6 32357890 PMC7193356

