# Supplementary material for: Implementing community case management of malaria: Stakeholder insights on advancing equitable access in Kilifi County
Source: PLOS Glob Public Health. 2026 Jul 6;6(7):e0006478. doi: 10.1371/journal.pgph.0006478 (PMC13336154; doi:10.1371/journal.pgph.0006478)
Supplement: S1 File — (DOCX) [file pgph.0006478.s001.docx]

**INFORMATION SHEET FOR COUNTY, SUB-COUNTY, FACILITY AND COMMUNITY LEVEL PARTICIPANTS**

**The Net Operational Research and Mapping Kilifi County, Kenya**

My name is {Insert name} a researcher working for The Net, a community based Malaria programme in Kilifi County. We are here conducting operational research focusing on Malaria preventive strategies and more specifically the impact of the Net Malaria programme in 7 sub-counties in Kilifi.

You are invited to take part in this operational research to investigate the prevalence of Malaria and assess the effectiveness of interventions for malaria control within an endemic region. Malaria remains a significant public health concern, particularly in Coastal, Kenya where it is endemic, leading to substantial morbidity and mortality rates. Understanding the prevalence of the disease and evaluating the efficacy of interventions such as insecticide-treated bed nets, indoor residual spraying, and antimalarial medications is crucial for guiding public health policies and implementing targeted strategies for malaria control. By conducting this study, we aim to contribute valuable insights into the status of Malaria within Kilifi and provide evidence-based recommendations for more effective control measures, ultimately working towards reducing the burden of this devastating disease on affected communities.

Before you decide to participate in this study, it is important that you read this form. The available alternatives and the right to withdraw your consent to participate at any time are described below. You have the right to ask questions at any time.

**Description of the study**

This is a mixed method study design.

W**here is the study taking place**?

This study will take place in Kilifi County. It targets communities and health facilities located in the following sub-counties: Kilifi North (Kilifi County Referral Hospital, Kiwandani and Kadzinuni Dispensary), Kilifi South (Pingilikani and Tunzanani), Ganze (Ganze Health Centre and Jaribuni Dispensary), Kaloleni (Mgamboni and Kinarani Dispensary), Rabai (Makanzani and Lenga Dispensary), Malindi (Kakuyuni and Gongoni Health Centre).

**How many participants does it involve?**

This study will involve about 150 participants from county, sub-county facility and community levels.

**How will they be selected?**

Participants in the key informant interviews and focus group discussions will be purposely selected from the County/ Sub-County Health Management Team, facility-in-charge or their representative, and community/ local government leaders such as location chiefs or ward administrators. Participants for the focus group discussions will be drawn from the health facility catchment areas and will be identified with the help of community health promoters.

**What does the study involve for those taking part?**

Each participant will be requested to provide written informed consent to provide data for the study. Individuals participating in focus group discussions and interviews will be requested to dedicate 45 minutes - 1 hour of their time to respond to a semi- structured set of question. Focus group discussions will be conducted in-person at a suitable location preferably a link health facility.

**Examinations in the context of the study**

If you accept to participate in the study, no invasive tests and examinations will be performed. The researcher will hold discussions in a location and time most convenient to you. If you do not want to answer any of the questions, you may say so and they will move on to the next question.

**Voluntary participation**

You participate entirely voluntarily in this study and you have the right to refuse to participate in the study. Your decision to participate in this study or not, will have no influence whatsoever on your work at this facility. You also have the right to stop your participation in the study at any time, even after you have signed the consent form. You do not have to give a reason for withdrawing your consent to participate. The withdrawal of your consent will not cause any disadvantage of loss of advantages. If you accept to participate in this study, you will receive this information form to keep it and you will be asked to sign the attached consent form.

**What are the risks and benefits of taking part?**

Risks are minimal in this study. There will be no physical risks to participating in this study. Where possible, all interviews will be conducted in person, else we will opt for telephone interviews. We will use audio recorders to capture the conversations during interviews and focus group discussions. During transcription, we will guard the confidentiality of each participant by using unique identifiers rather than actual names. We will de-identify any personal data before storing the data in password-protected computers and all paper forms will be stored in locked cabinets only accessible to the key study team. In addition to collecting data from the participants, we hope to have enriching discussions with community members from which they may learn more about this study and other aspects of malaria transmission interventions. We will reimburse travel costs to all participants attending the focus group discussions. We will also provide compensation to all key informant interviewees for their time.

**Advantages**

We cannot confirm that you will personally benefit directly from your participation in this study. If you consent to participate in this study, the information resulting from this study can contribute to better knowledge on effective ways of implementing Malaria interventions.

**Compensation**

All participants will receive compensation for their time and transport costs incurred to participate in the study according to local, ethically approved reimbursement rates.

**Protection of your private life**

Your identity and your participation to this study will be treated strictly confidential. You will not be identified by name or in any other identifying manner in files, results or publication concerning this study. Your identity remains secret since personal information will only be designated by a unique participant number (therefore coded).

The sponsor might use your personal information for other research purposes or in the context of healthcare related to data analysis and the presentation of research findings. Only coded personal information will be used for this purpose. Your personal information will be processed and analysed electronically (in the computer) or manually in order to determine the results of this study. You also have the right to request the researcher to give you access to your personal information and to correct it if necessary. The protection of personal data is legally established in Kenya’s data sharing and protection act.

**When does the study start and finish?**

The study kicks off on 1^st^ Jan 2025 and is estimated to be complete in July 2025.

**Sponsor of the study**

This study is funded by Italian Agency for Development and co-operation.

**Ethics committee**

This study has been reviewed by the Pwani University Ethics and Scientific Review Committee and the Kenyan National Commission for Science, Technology and Innovation (NACOSTI)

**Contact persons in the case of questions concerning the study**

If you think having incurred damage related to the study or if you have questions concerning the study or your rights as a participant, you can contact, now, during or after the study:

Study principal investigator: Dr. Simon Masha

Telephone: +254 723 750 847

Email: s.masha@pu.ac.ke

**CONSENT FORM FOR KEY INFORMANT INTERVIEWEES**

| **The Net Operational Research and Mapping Kilifi County, Kenya Lay explanation:** A study looking at approaches that have been taken to prevent Malaria in Kilifi county; and explore what challenges and opportunities have been experienced by different stakeholders. |
| --- |

| ***Part which is to be reviewed and signed by the interviewee (health worker / county management officer/health organization representative)***  I, (undersigned, name and first name) __________________ of designation _______________in ___________________confirm that I have been informed about the study and that I have received a copy of the information sheet and the consent form. I have read and understood the information. The researcher has given me sufficient information concerning the conditions and the length of the study. In addition, I have received sufficient time to consider the information and to ask questions, to which I have received satisfying answers.  – I have understood that I can put to a stop my participation in this study at any time after having informed the researcher about this, and that this decision will not cause any disadvantage.  – I agree with the use by the researchers of these coded data for other research purposes.  –   I consent voluntarily to participate in this study and to cooperate in all the questions. I am willing to give information concerning my practice, and interaction with clients.  – I agree that my clients and healthcare professionals involved in providing health care at this facility are informed about my participation in this study and as such:  1) Give consent / do not give consent* to take part in the study  2) Give consent/ do not give consent* to having the interview tape recorded  3) Give consent / do not give consent* to be anonymously quoted   (* Delete as appropriate).  I understand that I can change my mind at any stage and it will not affect me in any way.  ***Date: ___________                                                       Signature participant: _____________*** |
| --- |
| Part only designated to the investigator  I, undersigned, __________________________ confirm that I have informed, ________________ (full name of the participant) and that he/she has consented to participate in the study.  ***Date: _____________***  ***Signature investigator: _____________***___ |

**INTERVIEW GUIDE FOR IN-DEPTH INTERVIEWS (COUNTY AND SUB-COUNTY)**

The Net Operational Research and Mapping Kilifi County, Kenya

**Introduction**

The need to improve community awareness and health seeking behaviour for Malaria, and an aim to improve the quality of care offered at primary health care level has been an important part of health sector reforms in Kenya promoting equity in health and better functioning of health systems.

Thank you for making time to speak with us today

**Stakeholder mapping**

1. To begin, maybe you could tell us about yourself and your role as the (insert position of interviewee) in (insert designation)
2. What are your key roles and responsibilities?
   - 1. How long have you been carrying out these roles?
     2. What are your key roles and responsibilities related to the implementation of Malaria interventions?
     3. Did / do you receive training on how to carry out these roles?
     4. Are you still confident in carrying out these roles? Is there additional support you need?
3. Who else do you work closely with especially related to Malaria programmes and interventions? (develop organogram specific for Malaria programmes)
   - 1. How do you usually interact? (county, Partners and NGOs)
     2. Are there documents shared? Regular meetings?
     3. What are your thoughts about how they carry out their roles?

**Perceptions on the implementation of Malaria programmes**

1. What are your thoughts about the current state of Malaria in Kilifi county?
   - 1. Probe on: incidence, prevalence, community awareness: current interventions
     2. What are the current data management practices?
2. What Malaria prevention strategies have been implemented in your region; (Probe on LLINs, indoor residual spraying, mosquito repellents, intermittent prevention e.g. pregnant women) for each of these:
   - 1. What do you think has contributed to successful implementation?
     2. What are the challenges you have observed or experienced in the implementation of Malaria programmes?
     3. What do you consider to be good practice in caring for a client with Malaria - is this aligned with the current guidelines?
     4. What is the current status/availability of the Malaria vaccine?
3. Have you heard of the Net program?
   - 1. What do you know about it? – activities, training CCM
     2. What do you feel about its implementation to date?
     3. Achievements and successes
     4. Challenges faced / areas of improvement

**Perception on the impact of the NET**

1. How do you think the NET intervention has affected community knowledge, attitude and practices? Knowledge and understanding of malaria
   - 1. Awareness of risk, causes, transmission, symptoms
     2. Prevention of Malaria: Insecticide treated nets (ITNs), residual spraying, intermittent preventive treatment campaigns e.g. for pregnant women, chemoprophylaxis: repellents
     3. School education programmes, remember to also ask about traditional healers
2. What influence has the NET had on:
   - 1. Training of sub-county, facility and community level health providers?
     2. Community case management of uncomplicated malaria (screening diagnosis and treatment)?
     3. Referrals for complicated malaria? How do you feel about the care that your clients receive at the health facility (especially related to Malaria diagnosis and treatment)?
     4. Supervision of CHPs
     5. Malaria data management process
        - Check on the reporting process: Malaria daily activity register, MOH 516 Chalk Board, 513: HH register, MOH514: service delivery log book, community treatment and tracking register (data entry, accuracy, timeliness and completeness)
3. Are there any significant differences in the management of malaria cases where the NET programme is not present?

**Perception of quality of care offered at facilities**

1. How do you feel about the care that your clients receive at community level (especially related to community case management of Malaria)?
2. What is your perception of the quality of care offered at primary health facilities for clients with Malaria?
   - 1. What is the state of resources for primary health care facilities? e.g. medicine, equipment, non-pharmaceutical supplies, consultation rooms / space
     2. Perception of the characteristics of staff working in PHC facilities: training, qualifications, experience compensation, any incentives to work in rural areas, staff turn over
     3. How is the quality of care evaluated? e.g. supervision by the district level
3. Are there any other important activities or events that have happened or are happening that have an impact on the quality of care offered for clients with Malaria?
4. Do you have anything else you would like to speak about?
5. Do you have questions for us?

**Thank you so much for your time**

## INTERVIEW GUIDE FOR IN-DEPTH INTERVIEWS WITH FACILITY ICs

The Net Operational Research and Mapping Kilifi County, Kenya

**Introduction**

The need to improve community awareness and health seeking behaviour for Malaria, and an aim to improve the quality of care offered at primary health care level has been an important part of health sector reforms in Kenya promoting equity in health and better functioning of health systems.

Thank you for making time to speak with us today

**Stakeholder mapping**

1. To begin, maybe you could tell us about yourself and your role as the (insert position of interviewee) in (insert designation)
2. What are your key roles and responsibilities?
   - 1. How long have you been carrying out these roles?
     2. What are your key roles and responsibilities related to the implementation of Malaria interventions?
     3. Did / do you receive training on how to carry out these roles?
     4. Are you still confident in carrying out these roles? Is there additional support you need?
3. Who else do you work closely with especially related to Malaria programmes and interventions? (develop organogram specific for Malaria programmes)
   - 1. How do you usually interact? (county, Partners and NGOs)
     2. Are there documents shared? Regular meetings?
     3. What are your thoughts about how they carry out their roles?
4. Are there specific activities that you carry out related to Malaria prevention, diagnosis, treatment and follow-up? Please take me through them

Journey mapping from when they receive a client at the facility, health education, suspicion of / referred client with Malaria, what symptoms do they look out for (esp danger signs): clinical vs lab diagnosis, treatment and follow-up

Check on the reporting process: Malaria daily activity register, MOH 516 Chalk Board, 513: HH register, MOH514: service delivery log book, community treatment and tracking register (data entry, accuracy, timeliness and completeness)

- - 1. What is your opinion about this process
    2. How do you feel about the training you have received so far?
    3. What do you consider to be good practice in caring for a client with Malaria - is this aligned with the current guidelines
    4. Do you have all the resources you need?
    5. Do you have all the data/information you need?

1. Do you have activities related to the use of Insecticide Treated Nets at this facility?

Probe on:

- - 1. Access to ITNs, perceptions on the use of ITNs
    2. door to door CHP health education, school programmes

1. **Training:** How do you get all this information and what influences how you do the work that you have described?

- Probe on the NET training if not mentioned
- When was it done? Who was involved?
- What did it cover?
- Are there things you did not understand or would want to be included?

1. **Supportive supervision**
2. Do you get any supervision?
3. What happens during SS?
4. How do you feel about these sessions?
5. Are your questions/suggestions addressed?
6. **Client knowledge and understanding of Malaria**
7. Do you think that the clients you serve have heard about Malaria?
8. How would you gauge their knowledge on the risks of contracting Malaria? Are there particular groups that are at higher risk?
9. What do you think are the causes of Malaria? Do you think those views are shared by your peers and the community you serve? (Probe on reasons)
10. What are your thoughts about how Malaria is transmitted? Do you think your peers and the community you serve share those thoughts?(Probe on reasons)
11. Are you able to easily identify symptoms of Malaria? Which ones are you aware of? (probe on fever, feeling cold/chills, headache, loss appetite, body pain/joint) What are your opinions on the community understanding of the symptoms of Malaria?
12. How do you feel about the **care that your clients receive from CHPs** (especially related to Malaria diagnosis and treatment)?
    - 1. Probe on the referral process
      2. Do you get any feedback from the facility?
      3. How do you follow-up patients after care at the facility?
      4. Availability of resources
13. Are there any other important activities or events that have happened or are happening that have an impact on the quality of care offered for clients with Malaria offered in this primary health care facility?
14. Do you have anything else you would like to mention about Malaria prevention in your region?

Do you have questions for us?

**INTERVIEW GUIDE FOR IN-DEPTH INTERVIEWS FOR CHA**

The Net Operational Research and Mapping Kilifi County, Kenya

**Introduction**

The need to improve community awareness and health seeking behavior for Malaria, and an aim to improve the quality of care offered at primary health care level has been an important part of health sector reforms in Kenya promoting equity in health and better functioning of health systems.

**Stakeholder mapping**

For each of the key informants including members of the county and sub-county departments of health, health workers, community health promoters, local government representatives:

1. Maybe we could begin with you telling us a bit about yourself, what are your key roles and responsibilities as a CHA?
   1. What are your key roles and responsibilities related to the implementation of Malaria interventions?
   2. How long have you been carrying out these roles?
   3. What is your background?
   4. did you receive training on how to carry out those roles?
2. How confident are you about carrying out your roles, are there things you would need?
3. Who do you work closely with especially related to Malaria programs and interventions?
   1. How do you usually interact?
   2. Are there documents shared? Regular meetings?
   3. What are your thoughts about how they carry out their roles? Perceptions on the implementation of Malaria programs
4. What are your thoughts about the current state of Malaria in Kilifi county and in your specific region?
   1. Number of cases: incidence and prevalence
   2. Community awareness: current interventions
   3. What Malaria prevention strategies have been implemented in your region; (Probe on LLINs, indoor residual spraying, mosquito repellants, intermittent prevention e.g. pregnant women) for each of these:
      1. What do you think has contributed to successful implementation?
      2. What are the challenges you have observed or experienced in the implementation of Malaria programs?
5. Have you heard **of the Net program?**
   1. What do you know about it?
   2. What are the key supported activities?
   3. How do you feel about its implementation to date?
      1. Achievements and successes
      2. Challenges faced / areas of improvement
      3. How has it changed the management of Malaria in this community?

**Perception on community Knowledge attitudes and practices**

1. Knowledge and understanding of malaria
   1. What do you think CHPS understand about?
      1. What Malaria is?
      2. How would you gauge their knowledge on the risks of contracting Malaria? Are there particular groups that are at higher risk?
      3. What do you think they understand as the causes of Malaria?
      4. How Malaria is transmitted?
      5. Prevention strategies
         1. ITNs
         2. Spraying
         3. Preventive treatment campaigns for women
         4. Chemoprophylaxis
      6. Are they able to easily identify symptoms of Malaria?
   2. What do you think about the community perception towards Malaria
      1. For each of the mentioned strategies, do you think the community is aware? how do you think the community has responded - do they feel it is important?

**Screening, diagnosis and treatment of Malaria**

1. What do you think is good practice in how CHPs should take care of clients with Malaria?
   1. Journey mapping from when they visit a home, health education, suspicion of a client with Malaria, what symptoms do they look out for screening (RDTs), identification of danger signs, testing (procedure and interpretation, storage of RDTs), treatment including explanation of dosage and schedule for uncomplicated malaria, referral, follow-up (3 days), reporting (referral form)
   2. Do they have all the resources they need? (RDT kit, Non sterile gloves, Dry cotton wool or cotton gauze, Marker pens, Clock or timer, Sharps disposal container, Biosafety kit
   3. AL med

**Supportive supervision**

1. How do you offer support to CHPs?
   1. What happens during SS?
   2. How do you feel about these sessions?
   3. Probe on data management and forms filled
2. Do you get any supervision from the facility or sub-county?
   1. Are your questions/suggestions addressed?

**Perception of quality of care offered at facilities**

1. How do you feel about the care that your clients receive at community level (especially related to Malaria diagnosis and treatment)?
2. What is your perception of the quality of care offered at primary health facilities?
3. What is the state of resources for primary health care facilities? e.g. medicine, equipment, non-pharmaceutical supplies, consultation rooms / space
4. Perception of the characteristics of staff working in PHC facilities: training, qualifications, experience compensation, any incentives to work in rural areas, staff turn over
5. How is the quality of care evaluated? e.g. supervision by the district level
6. Are there any other important activities or events that have happened or are happening that have an impact on the quality of care offered for clients with Malaria offered in this primary health care facility?
7. Do you have anything else you would like to mention about Malaria prevention in your region?
8. Do you have questions for us?

**INTERVIEW GUIDE FOR IN-DEPTH INTERVIEWS WITH CHPs**

The Net Operational Research and Mapping Kilifi County, Kenya

**Introduction**

The need to improve community awareness and health seeking behaviour for Malaria, and an aim to improve the quality of care offered at primary health care level has been an important part of health sector reforms in Kenya promoting equity in health and better functioning of health systems.

Thank you for making time to speak with us today

**Stakeholder mapping**

1. To begin, maybe you could tell us about yourself and your role as the (insert position of interviewee) in (insert designation)
2. What are your key roles and responsibilities?
   - 1. How long have you been carrying out these roles?
     2. What are your key roles and responsibilities related to the implementation of Malaria interventions?
     3. Did / do you receive training on how to carry out these roles?
     4. Are you still confident in carrying out these roles? Is there additional support you need?
3. Who else do you work closely with especially related to Malaria programmes and interventions? (develop organogram specific for Malaria programmes)
   - 1. How do you usually interact? (county, Partners and NGOs)
     2. Are there documents shared? Regular meetings?
     3. What are your thoughts about how they carry out their roles?

Perception on community Knowledge attitudes and practices

*(these questions will also be the particular focus of community member FGDS)*

1. **Knowledge and understanding of Malaria**
2. Do you think that the clients you serve have heard about Malaria?
3. How would you gauge their knowledge on the risks of contracting Malaria? Are there particular groups that are at higher risk?
4. What do you think are the causes of Malaria? Do you think those views are shared by your peers and the community you serve? (Probe on reasons)
5. What are your thoughts about how Malaria is transmitted? Do you think your peers and the community you serve share those thoughts?(Probe on reasons)
6. Are you able to easily identify symptoms of Malaria? Which ones are you aware of? (probe on fever, feeling cold/chills, headache, loss appetite, body pain/joint) What are your opinions on the community understanding of the symptoms of Malaria?

**Prevention of Malaria**

1. Are you aware of any Malaria prevention strategies (Probe on intervention, target population, stakeholder support, awareness campaigns and training and overall perception of progress to date)
2. Insecticide treated netsTNs
3. Residual spraying
4. Intermittent preventive treatment campaigns e.g. for pregnant women
5. Chemoprophylaxis: repellants

For each of the mentioned strategies, do you think the community is aware? how do you think the community has responded - do they feel it is important? Are there any other Malaria prevention strategies?

**Screening, diagnosis and treatment of Malaria**

1. (For CHPs) Are there specific activities that you carry out related to Malaria screening, diagnosis and treatment follow-up? Please take me through them
   - 1. Journey mapping from when they visit a home, health education, suspicion of a client with Malaria, what symptoms do they look out for screening (RDTs), identification of danger signs, testing (procedure and interpretation, storage of RDTs), treatment including explanation of dosage and schedule for uncomplicated malaria, referral, follow-up (3 days), reporting (referral form)
     2. What is your opinion about this process?
     3. Did you receive any training related to this?
     4. How do you feel about the training you have received so far, probe on: -knowledge of populations at risk of getting malaria, transmission, diagnosis and treatment; external influencing factors: SES, climate change, traditional beliefs and practices
     5. Do you have all the resources you need? (RDT kit, Non sterile gloves, Dry cotton wool or cotton gauze, Marker pens, Clock or timer, Sharps disposal container, Biosafety kit, AL med
     6. Data reporting and procedures: which forms do you fill in and to whom and when do you submit them ?
2. **Training:** How do you get all this information and what influences how you do the work that you have described?

- Probe on the NET training if not mentioned
- When was it done? Who was involved?
- What did it cover?
- Are there things you did not understand or would want to be included?

1. **Supportive supervision**
2. Do you get any supervision from CHEW?
3. What happens during SS?
4. How do you feel about these sessions?
5. Are your questions/suggestions addressed?
6. How do you feel about the **care that your clients receive at the health facility** (especially related to Malaria diagnosis and treatment)?
   - 1. Probe on the referral process
     2. Do you get any feedback from the facility?
     3. How do you follow-up patients after care at the facility?
     4. Availability of resources
7. Are there any other important activities or events that have happened or are happening that have an impact on the quality of care offered for clients with Malaria offered in this primary health care facility?
8. Do you have anything else you would like to mention about Malaria prevention in your region?
9. Do you have questions for us?

### FOCUS GROUP DISCUSSION GUIDE FOR COMMUNITIES

**The Net Operational Research and Mapping Kilifi County, Kenya**

Welcome and thank you for volunteering to participate in this focus group. Your point of view is important and we appreciate your time. I would now like to give you some information about our discussion today (go through the information sheet and consent process above)

**Purpose**

The purpose of this operational research is to engage the community in a focus group discussion (FGD) with community members to find out the knowledge, attitude, perceptions, practices surrounding Malaria and preventive strategies. This is important for us to understand what community members already know and think about Malaria and the prevention strategies that have been implemented including the Net project. This will be important to the community as findings from this study will be shared to the county and its implementing partners and may contribute to more informed planning and decision making; in a way that adapts health messages and activities to better respond to the actual needs and socio-cultural context in the community. This FGD will also help us better understand people’s risk perceptions, health behaviors and practices that are being appropriate or could instead be increasing their risk of contracting Malaria.

**Go through the participant information sheet (sample shared above will be customized for a group audience)**

| **Date of discussion:** | **Moderator:** |
| --- | --- |
| **Venue:** | **Note-taker:** |
| **Time start:** | **No. Participants at start:** |
| **Time stop:** | **No. Participants at stop:** |

Written group consent for FGD

| We have had the study explained to us by ____________________from __________________. We have understood all that has been read/explained. We were given the opportunity to seek clarification and had our questions answered satisfactorily.    We hereby:  1) Give consent / do not give consent* to take part in the study  2) Give consent / do not give consent* to having the interview tape recorded   (* Delete as appropriate).  We understand that we can change our minds at any stage and it will not affect us in any way.    Signature of group representative _______________________ Date: ______________________    Name initials _____________________________     Facility Name: ______________________ |
| --- |

| **PARTICIPANT DEMOGRAPHIC DATA** | | | | | | | |
| --- | --- | --- | --- | --- | --- | --- | --- |
| **Participant** | **Gender** | **Age** | **Education (Highest level)** | **Occupation** | **No. of HH members** | **No. of sleeping spaces** | **No. of nets/ did you sleep under a net last night** |
| **1** |  |  |  |  |  |  |  |
| **2** |  |  |  |  |  |  |  |
| **3** |  |  |  |  |  |  |  |
| **4** |  |  |  |  |  |  |  |
| **5** |  |  |  |  |  |  |  |
| **6** |  |  |  |  |  |  |  |
| **7** |  |  |  |  |  |  |  |
| **Moderator’s remarks:** | | | | | | | |

FGD GUIDING QUESTIONS/TOPICS

1. As we talked about before, we are here to talk with you about your personal and healthcare experiences especially related to Malaria
2. Have you heard about Malaria? Would anyone like to share what they know about Malaria?
3. How do you feel about the risks of contracting Malaria?  Are there particular people that are at higher risk?
4. What do you think are the causes of Malaria? (Probe on reasons) where did you get that information from?
5. How would you know What are your thoughts about how Malaria is transmitted? (Probe on reasons and source of information)
6. How would you know if you or someone in your family has Malaria? Which symptoms are you aware of? (probe on fever, feeling cold/chills, headache, loss appetite, body pain/joint)
7. Prevention of Malaria
8. What do you do to protect yourself and your family from getting Malaria?
   1. ITNs
   2. Residual spraying
   3. Chemoprophylaxis
9. How do you feel about these methods - do you understand them? are they important? Are they available to you?

**Questions particular to ITNs**

3. Access to ITNs

1. How many of you own a net? Where did you get it/them from?
2. How long have you had it/them? - what's the current condition?
3. What type of net? Long-lasting insecticide-treated nets (LLINs) and conventional ITNs (cITNs)
4. Are you able to get more?
5. When and how would you replace an old/damaged one?

4. Use of ITNs

1. How often do you sleep under a net?
   1. Consistently
   2. Sometimes: probe on determinants e.g. rainy season, traveling, occupation, SES factors
   3. Is it the same for the other members of the household? - probe on children under 5 and pregnant women
   4. How do you usually place the net
   5. What makes you influence whether you sleep under a net or not?
2. What motivates people to keep sleeping under nets? What do you see as the benefits of sleeping under a treated net?
3. Are there any challenges that make it difficult to consistently sleep under a net?

**Perceived quality of care given by CHPs**

5. Have any of you ever been tested or treated for Malaria? who would like to share what the experience was like.

6. Did you receive any intervention/care from a community health promoter? What was it and how did the experience make you feel? (Probe on home visit-referral-facility and follow-up experiences) special attention to:

1. Health education on Malaria
2. Rapid diagnostic tests (RDTs)
3. Treatment of uncomplicated malaria
4. Understanding and adherence to treatment
5. Referral process
6. Facility visit, diagnosis and treatment
7. Over-the-counter treatment
8. Follow up

**Perceived quality of care given at health facilities**

7. What are your opinions of the quality of care offered in primary health care facilities / are you satisfied with the services?

1. What about Malaria services: lab diagnosis and medication
2. What do they like? What do they not like? (write out opinions on a flip chart)

8. What do you think of the interactions with health workers in primary health facilities? Especially when you or a member of your family has Malaria?

1. How confident are you after leaving the facility?

9. Do you face any challenges in accessing health services at primary health care level (financial, distance from home, drugs and medication and other indirect costs)

1. Any suggestions on how these challenges can be handled?

10. Are there any other issues regarding Malaria and Malaria prevention that you would like to discuss?

11. Do you have any questions for us?

Thank you so much for participating in today's discussion, please remember to pick your transport reimbursement before you leave.
