# Supplementary material for: Implementing community case management of malaria: Stakeholder insights on advancing equitable access in Kilifi County
Source: PLOS Glob Public Health. 2026 Jul 6;6(7):e0006478. doi: 10.1371/journal.pgph.0006478 (PMC13336154; doi:10.1371/journal.pgph.0006478)
Supplement: S2 File — (DOCX) [file pgph.0006478.s002.docx]

**S2. Treatment schedules and list of documents - extracts from CHP training guidelines**


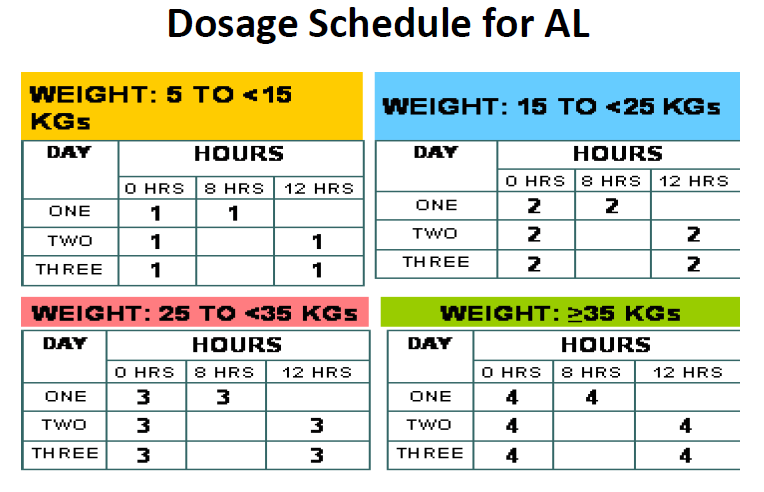

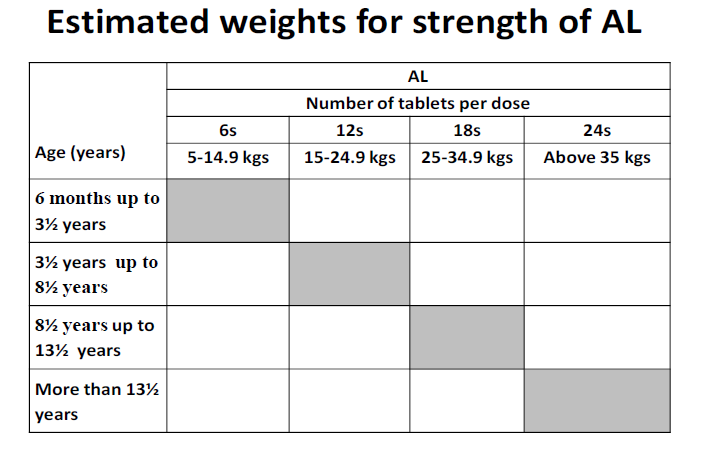


**Documenting malaria indicators**

- Facility records
  - Malaria Commodities Daily Activity Register (MOH 705 / 204): for each of the registers, there were registers for children under 5 years and persons over the age of 5 years. MOH 204 additionally had the names of the patients.
  - CHEW monthly summary data (MOH 515): number of malaria cases tested and treated for under 5 and persons over 5 years
  - Chalk board (MOH 516)
  - Community health unit monthly summary report for malaria commodities (MOH 748)
  - Service Delivery Log Book (MOH 514): number of malaria cases
  - Facility consumption data report and request (MOH 643): RDTs issued and used
  - Weekly epidemic monitoring form (MOH 505 - IDSR): suspected, tested and confirmed cases; and deaths from Malaria
  - Health facility monthly summary report for Malaria commodities (MOH 743): artemether‐lumefantrine bands quantities, usage and expiry
- CHP records
  - CHP monthly data summary (number of malaria cases tested and treated)
  - Community treatment and tracking register (MOH 521): RDT test results and severe cases
  - CHP daily activity register for Malaria commodities (MOH 648): reporting on RDTs and ALs usage
  - Household register (MOH 513)
  - Referral form (MOH 100)
