# Supplementary material for: Implementing community case management of malaria: Stakeholder insights on advancing equitable access in Kilifi County
Source: PLOS Glob Public Health. 2026 Jul 6;6(7):e0006478. doi: 10.1371/journal.pgph.0006478 (PMC13336154; doi:10.1371/journal.pgph.0006478)
Supplement: Attachment — Submitted filename: Waweru et al-2025-review01.docx [file pgph.0006478.s003.docx]

Review of Waweru et al

General comments

This reviewer commends the authors for a thoughtfully conceived and carefully implemented study. I particularly liked the conceptual framework, which articulated the assumptions (often not realized) that might have created a well-functioning CHP program. This reviewer also appreciated the limitations section, which appropriately noted that the program operated under time-limited external funding, which was not generalizable.

This reviewer would encourage the authors to elaborate in the discussion section on how the findings from their study could be useful in the context of most health facilities in Kenya. Resources are often inadequate. Regular health staff are human beings with their strengths, limitations, transportation constraints, and outside responsibilities.

While the health facility data could not be linked directly to the CHPs’ data, could a comparative analysis between the 10 treatment and the 4 control sites indicate whether and by how much the CHPs reduced the workload on health facilities and the resulting savings.

My main recommendation is that the authors suggest ways that the CHP program might be adapted to deliver as many benefits as possible within the resources that are actually available in a typical control facility. Could the system be designed to recognize that remuneration for CHWs is often inadequate and sometimes unavailable? Could CHPs be given non-monetary benefits that could always be guaranteed? Examples include certificates they could put in their kit or display in their house, a pin they could affix to their clothing while working, and priority for being seen when they attend the base primary care facility. For allowances (such as stipends and airtime), could the best CHPs be prioritized. Priorities could be based on activity reports and, perhaps, feedback from their clients who attend health facilities.

Knowing that CHPs and formal health facilities may have inadequate supplies of tests, medications, and staff, could CHWs be taught how to be careful stewards of these scarce resources. Thus, they might be taught to base their decision about whom to test for suspected malaria based on risk (age and symptoms). Similarly, they might be taught for patients who test negative for malaria to offer paracetamol and similarly decide whether to refer patients to a health facility immediately based on risk (age and symptoms). Those not referred immediately should be given clear instructions regarding how long to wait and signs to look for to decide whether they should visit a health facility. For example, if the illness proved to be a bacterial infection, antibiotic treatment would be effective. If it were a viral illness, a treatment such as paracetamol (which a CHP could provide) mght be the only option. If the illness were a symptom of some other disease, the diagnostic skills of the health center staff, and potentially further referrals, would be helpful.

Likewise, the CHPs might focus more efforts on measures that do not require new resources, such as reinforcing recommendations on malaria prevention. These might be geared primarily towards pregnant women and young children, who are most vulnerable to adverse outcomes from malaria. This might entail observing malaria nets, asking about recent use, and refreshing instructions for high-risk community members (e.g. pregnant women and young children).

Finally, suppose the rural health facilities could obtain limited additional resources (e.g. 10% more). The authors would do a service by indicating which malaria-related services would be the highest priority, the expected improvements in health outcome (quantified if possible) and the reasons behind their priorities.

Specific comments

Line 51: It would be helpful if the authors could update the global number to the latest available data.

Line 54: I would suggest writing Malaria in lower case

Line 56: Could you consider rewording “Lack of cooperation from the communities” as it reads like a version of “blame the victim.”

Line 137: Change “The study…” to “The study’s…”

Line 195: Change “majority of…” to “the majority of…”

Line 198. In percentage, round to the nearest whole percent, given the small sample size.

Line 198: The table would be clearer to this reviewer with the following editorial changes:

Label the first column “Characteristic,” the second column “Category”, the third column “Number of participants” and fourth column “Percentage or mean.” Then divide the table into three panels, with each panel labeled a centered heading. The first subpanel might be labeled “CHPs (n=42).” the second “In-charges (n=14)”, and the third “Community members (n=76)”. In the data in the last column, use a percent sign for the percentage entries to distinguish them from averages.

I think that the “Summary of data collected” could be added to the text for any important items not already stated, as it does not fit the format above.

Line 241. I think it better to describe the rate of 21.1% as the “positivity rate.” By contrast, I would reserve “prevalence” for the results of a population-based testing. In this study, all persons tested likely had a symptom or suspicion of possible malaria. Also change “>” to “≥” to clarify that those age 5 were in the upper category.

Line 250. The table would be strengthened by adding the following additional columns: Estimated pop.<5 yrs, Est. pop ≥ 5 yrs, % of pop <5 tested, % of pop ≥5 tested. These columns would give useful population-based data. The supplement could indicate how the authors used existing census information and updates to estimate the populations.

Line 345. Those with negative test results were referred to health facilities. It would seem helpful if CHPs could distinguish those with negative tests between severe or at risk (based on symptoms, age and pregnancy) and not at risk. Those not at risk might have self-limiting illnesses. The authors might comment on the appropriateness of the guidelines in S2 in view of the resource constraints.

Line 508. The overall prevalence … was 21.1%. This reviewer disagrees with the interpretation. The statistic is the positivity rate. It depends on the relationship between malaria and other conditions giving sufficient suspicion of malaria to warrant a test.

Line 513. Capitalize all words in the title: “World Malaria Report”

Line 516. Please clarify the numerator and denominator of the 17% statistic and present findings from control sites to guide readers in the interpretation.

Line 547. Add apostrophe: “…CHPs’ abilities…”
