# Supplementary material for: Implementing community case management of malaria: Stakeholder insights on advancing equitable access in Kilifi County
Source: PLOS Glob Public Health. 2026 Jul 6;6(7):e0006478. doi: 10.1371/journal.pgph.0006478 (PMC13336154; doi:10.1371/journal.pgph.0006478)
Supplement: Attachment — Submitted filename: Response to editorial and reviewer comments PGPH-D-25-03246.docx [file pgph.0006478.s004.docx]

## PGPH-D-25-03246

### Response to PGPH Journal Requirements:

1. Please provide a detailed online Financial Disclosure statement. This is published with the article. It must therefore be completed in full sentences and contain the exact wording you wish to be published.

a) State the initials, alongside each funding source, of each author to receive each grant. For example: “This work was supported by the National Institutes of Health (####### to AM; ###### to CJ) and the National Science Foundation (###### to AM).”

b) State what role the funders took in the study. If the funders had no role in your study, please state: “The funders had no role in study design, data collection and analysis, decision to publish, or preparation of the manuscript.”

For more information, please go to our submission guidelines:
<https://journals.plos.org/globalpublichealth/s/submission-guidelines#loc-financial-disclosure-statement>

**Response on the financial disclosure statement:** The updated financial disclosure statement now reads “This work was supported by the Italian Agency for Development Cooperation (AICS) (Project code: 012596/01/4) awarded to GM (Dr. Gianfranco Morino, World Friends Regional Coordinator). World Friends launched a tender procedure for project evaluation (Tender Ref. WF-K-2024-13) won by SM (Dr. Simon Masha – Think Health Consultants Limited / Pwani University).”

**Response on the role of funders:** “The funders had no role in study design, data collection and analysis, decision to publish, or preparation of the manuscript.”

2. Please ensure that the funders and grant numbers match between the Financial Disclosure field and the Funding Information tab in your submission form. Note that the funders must be provided in the same order in both places as well.

**Response:** The funders and grant numbers match between the Financial Disclosure field and the Funding Information tab in our submission form.

3. Please update your online Competing Interests statement. If you have no competing interests to declare, please state: "The authors have declared that no competing interests exist."

**Response on Competing Interests statement:** text on the online Competing Interests statement now reads "The authors have declared that no competing interests exist."

4. We note that you have indicated that there are restrictions to data sharing for this study. For studies involving human research participant data or other sensitive data, we encourage authors to share de-identified or anonymized data. However, when data cannot be publicly shared for ethical reasons, we allow authors to make their data sets available upon request. For information on unacceptable data access restrictions, please see <http://journals.plos.org/globalpublichealth/s/data-availability#loc-unacceptable-data-access-restrictions>.

Before we proceed with your manuscript, please address the following prompts:
 
a) If there are ethical or legal restrictions on sharing a de-identified data set, please explain them in detail (e.g., data contain potentially identifying or sensitive patient information, data are owned by a third-party organization, etc.) and who has imposed them (e.g., a Research Ethics Committee or Institutional Review Board, etc.). Please also provide contact information for a data access committee, ethics committee, or other institutional body to which data requests may be sent.

b) If there are no restrictions, please upload the minimal anonymized data set necessary to replicate your study findings to a stable, public repository and provide us with the relevant URLs, DOIs, or accession numbers. Please see <http://www.bmj.com/content/340/bmj.c181.long> for guidelines on how to de-identify and prepare clinical data for publication. For a list of recommended repositories, please see <https://journals.plos.org/globalpublichealth/s/recommended-repositories>. You also have the option of uploading the data as Supporting Information files, but we would recommend depositing data directly to a data repository if possible.

Please update your Data Availability statement in the submission form accordingly."

**Authors’ response:** Our data availability statement now reads:

This research article provides both qualitative and quantitative data for discussion. The quantitative demographic data of participants (de-identified), and data on malaria indicators for Kilifi, Kenya is now uploaded on Zenodo repository and can be accessed through this link <https://zenodo.org/records/19925126> .

For our analysis we have utilized data from the following (MOH 706) at the 10 facilities implementing CCMm in the 5 sub-counties. Specifically, for table 2 we have utilized the following:

- MOH 706 Rev 2020_Malaria Rapid diagnostic Tests (Under five years) Total Exam
- MOH 706 Rev 2020_Malaria Rapid diagnostic Tests (Under five years) Number Positive
- MOH 706 Rev 2020_Malaria Rapid diagnostic test (5 years and above) Total Exam
- MOH 706 Rev 2020_Malaria Rapid diagnostic test (5 years and above) Number Positive

Qualitative interview data remains restricted because data was collected in only 14 facilities, (and the 10 intervention facilities are well known). This means that complete interviews even when de-identified can be easy to assign to particular CHPs or in-charges. Subsequently, these can only be shared by making an application requesting access to the anonymized interview transcripts and outlining the purpose of accessing the data to the Pwani University ethics committee [puerc@pu.ac.ke](mailto:puerc@pu.ac.ke), the data governance unit [data-feedback@pu.ac.ke](mailto:data-feedback@pu.ac.ke) , and World Friends Kilifi, Kenya [info@world-friends.org](mailto:info@world-friends.org).

5. Please upload your main article file as a .doc, .docx or .rtf file.

With regards to this, two files have been uploaded

- Unmarked Updated manuscript
- Updated manuscript with tracked changes

6. Some material included in your submission may be copyrighted. According to PLOS’s copyright policy, authors who use figures or other material (e.g., graphics, clipart, maps) from another author or copyright holder must demonstrate or obtain permission to publish this material under the Creative Commons Attribution 4.0 International (CC BY 4.0) License used by PLOS journals. Please closely review the details of PLOS’s copyright requirements here: PLOS Licenses and Copyright. If you need to request permissions from a copyright holder, you may use PLOS's Copyright Content Permission form.

Please respond directly to this email or email the journal office and provide any known details concerning your material's license terms and permissions required for reuse, even if you have not yet obtained copyright permissions or are unsure of your material's copyright compatibility.

Potential Copyright Issues:

Figure 1: please (a) provide a direct link to the base layer of the map (i.e., the country or region border shape) and ensure this is also included in the figure legend; and (b) provide a link to the terms of use / license information for the base layer image or shapefile. We cannot publish proprietary or copyrighted maps (e.g. Google Maps, Mapquest) and the terms of use for your map base layer must be compatible with our CC-BY 4.0 license.

Note: if you created the map in a software program like R or ArcGIS, please locate and indicate the source of the basemap shapefile onto which data has been plotted.

If your map was obtained from a copyrighted source please amend the figure so that the base map used is from an openly available source. Alternatively, please provide explicit written permission from the copyright holder granting you the right to publish the material under our CC-BY 4.0 license.

Please note that the following CC BY licenses are compatible with PLOS license: CC BY 4.0, CC BY 2.0 and CC BY 3.0, meanwhile such licenses as CC BY-ND 3.0 and others are not compatible due to additional restrictions.

If you are unsure whether you can use a map or not, please do reach out and we will be able to help you. The following websites are good examples of where you can source open access or public domain maps: 
* U.S. Geological Survey (USGS) - All maps are in the public domain. ([http://www.usgs.gov](http://www.usgs.gov/)) 
* PlaniGlobe - All maps are published under a Creative Commons license so please cite “PlaniGlobe, [http://www.planiglobe.com](http://www.planiglobe.com/), CC BY 2.0” in the image credit after the caption. (<http://www.planiglobe.com/?lang=enl>) 
* Natural Earth - All maps are public domain. (<http://www.naturalearthdata.com/about/terms-of-use/>)

Figure 1. The figure now is in the format requested by the journal, please see attached details on GADM and CC BY 4.0. The new caption for figure 1 is in **line 123-129:**

**Figure 1.** Map showing the location of fourteen health facilities located in seven sub-counties. This include ten health facilities from five sub counties: Ganze (Ganze, Jaribuni), Kaloleni (Kinarani, Mgamboni), Kilifi North (Kiwandani, Kadzinuni), Kilifi South (Tunzanani, Pingilikani), and Rabai (Lenga, Makanzani) where CCMm was implemented. Data was also collected from four primary health facilities in Malindi sub-county (Madunguni, Baolala) and Magarini sub-county (Gongoni, Baricho) where CCMm was not implemented. GADM, <https://gadm.org/maps/KEN.html>, CC BY 4.0 (<https://gadm.org/license.html>).

7. If the reviewer comments include a recommendation to cite specific previously published works, please review and evaluate these publications to determine whether they are relevant and should be cited. There is no requirement to cite these works unless the editor has indicated otherwise.

N/A

## Reviewer's Responses to Questions

Reviewer #1: Review of Waweru et al.

General comments

This reviewer commends the authors for a thoughtfully conceived and carefully implemented study. I particularly liked the conceptual framework, which articulated the assumptions (often not realized) that might have created a well-functioning CHP program. This reviewer also appreciated the limitations section, which appropriately noted that the program operated under time-limited external funding, which was not generalizable.

**Authors’ response:** Thank you Prof. Shepard for your insightful and encouraging comments, we appreciate them.

This reviewer would encourage the authors to elaborate in the discussion section on how the findings from their study could be useful in the context of most health facilities in Kenya. Resources are often inadequate. Regular health staff are human beings with their strengths, limitations, transportation constraints, and outside responsibilities.

**Authors’ response: we have added this text to the discussion**

Line 603-611: CCM is specifically more relevant in a context with constrained resources because it acknowledges the key constraints in access to health facility services and helps ease the high facility health worker workload by shifting tasks like testing and treating uncomplicated malaria from high volume health facilities to CHPs in community health units, making the health system more resilient. Our study findings corroborate these benefits in the expansion of access to malaria testing and treatment, as well as added benefits in improving CHP competency and community trust. Evidence from other Kenyan counties also shows that CHPs have been able to deliver effective care for uncomplicated malaria and improve efficiency of referral for clients with severe cases of malaria using already existing human resources (CHPs), with additional training.

Note: we assume that when you mention regular health staff you mean health workers working at health facilities? Challenges faced by community-based health workers (called CHPs in Kenya) are already widely included in the discussion.

While the health facility data could not be linked directly to the CHPs’ data, could a comparative analysis between the 10 treatment and the 4 control sites indicate whether and by how much the CHPs reduced the workload on health facilities and the resulting savings.

**Authors’ response:** We only collected data from 4 control sites, against 10 intervention sites hence the statistical power and balance may be limited to match the facilities and perform comparative analysis between the two groups. In this manuscript, we can only suggest that savings are realized by utilizing CHPs to provide malaria diagnosis and treatment to patients outside the health facility set-up. Since the CHPs will be performing tasks which in health facilities would be done by several carder of staff involved in malaria case management, also savings on the part the community members in terms of time and transportation costs to the facility.

Our research did not set up to collect data such as:

- Cadre of staff involved in malaria case management, for example nurse, clinical officer, laboratory staff, pharmacy staff, records clerk
- Number of staff per cadre at each facility
- Monthly salary or total employment cost per cadre
- Average time spent per malaria/febrile patient by each cadre
- Registration time
- Consultation time
- RDT testing time
- Dispensing/counselling time
- Time spent on referrals and follow-up
- Number of working days and hours per month
- Time spent by patients
- Transportation costs for patients

Considering the above indicators, we cannot provide a response to the savings in time or cost. We have added this to the limitations of the study and will recommend further time-driven activity-based costing research and analysis.

My main recommendation is that the authors suggest ways that the CHP program might be adapted to deliver as many benefits as possible within the resources that are actually available in a typical control facility. Could the system be designed to recognize that remuneration for CHWs is often inadequate and sometimes unavailable? Could CHPs be given non-monetary benefits that could always be guaranteed? Examples include certificates they could put in their kit or display in their house, a pin they could affix to their clothing while working, and priority for being seen when they attend the base primary care facility. For allowances (such as stipends and airtime), could the best CHPs be prioritized. Priorities could be based on activity reports and, perhaps, feedback from their clients who attend health facilities.

**Author response:** This has been included in the discussion see paragraph on addressing systemic and resource related barriers line 656

Knowing that CHPs and formal health facilities may have inadequate supplies of tests, medications, and staff, could CHWs be taught how to be careful stewards of these scarce resources. Thus, they might be taught to base their decision about whom to test for suspected malaria based on risk (age and symptoms). Similarly, they might be taught for patients who test negative for malaria to offer paracetamol and similarly decide whether to refer patients to a health facility immediately based on risk (age and symptoms). Those not referred immediately should be given clear instructions regarding how long to wait and signs to look for to decide whether they should visit a health facility. For example, if the illness proved to be a bacterial infection, antibiotic treatment would be effective. If it were a viral illness, a treatment such as paracetamol (which a CHP could provide) might be the only option. If the illness were a symptom of some other disease, the diagnostic skills of the health center staff, and potentially further referrals, would be helpful.

**Author’s response**: In the results CHPs indicated that they tested for malaria based on signs and symptoms and all other cases were referred immediately to the health facility for diagnosis and treatment. This is in accordance with the additional training and practice they received in order to offer CCMm. CHPs do not yet have training in the diagnosis and treatment of other illnesses. They are allowed to give paracetamol (which they do when needed) but refer the client immediately. They cannot therefore tell the client to wait, or complications to look out for, it would be outside the scope of CHP practice. They get this service from their nearest health facility. Patients who test negative for malaria at the health facility follow the facility procedure and get treatment from facility-based health workers who have more training.

Likewise, the CHPs might focus more efforts on measures that do not require new resources, such as reinforcing recommendations on malaria prevention. These might be geared primarily towards pregnant women and young children, who are most vulnerable to adverse outcomes from malaria. This might entail observing malaria nets, asking about recent use, and refreshing instructions for high-risk community members (e.g. pregnant women and young children).

**Author’s response:** Outlined in contextual activities outside of CCMm

Finally, suppose the rural health facilities could obtain limited additional resources (e.g. 10% more). The authors would do a service by indicating which malaria-related services would be the highest priority, the expected improvements in health outcome (quantified if possible) and the reasons behind their priorities.

**Authors’ response:** This is beyond the scope of the data collected in this study as prioritization needs to be based on both structured (epidemiological data and community needs assessment, scope of work/workload, compensation and incentives, training and retention, vulnerable populations) and unstructured (acceptability and cultural relevance, health system integration) measurable outcomes etc. would be a great recommendation for future implementation cycles.

Specific comments

Line 51: It would be helpful if the authors could update the global number to the latest available data.

**Authors’ response** this has now been added and reference updated to read “According to the World Malaria Report for 2025, there were an estimated 282 million cases of malaria globally in 2024, with an incidence of 64 cases per 1000 population at risk. This represented an increase of 9 million cases from the previous year and a rise in incidence from 62.7 cases per 1000 population at risk in 2023.  The WHO African Region continues to carry the heaviest burden of malaria, accounting for an estimated 94% of malaria cases and 95% of malaria deaths worldwide in 2024; 75% of all deaths in this region were among children aged under 5 years old (1)”.

Line 54: I would suggest writing Malaria in lower case

**Authors’ response:** all changed expect in reference to documents e.g. Kenya Malaria Strategy, Kenya Malaria Indicator survey, World Malaria Report … or when it is the first word in a sentence.

Line 56: Could you consider rewording “Lack of cooperation from the communities” as it reads like a version of “blame the victim.”

**Authors’ response:** we replaced "lack of corporation from communities" with “challenges in community engagement and low levels of community participation” to make the comment less victim blaming. Now Line 58

Line 137: Change “The study…” to “The study’s…”

**Authors’ response:** changed, now line 140

Line 195: Change “majority of…” to “the majority of…”

**Authors’ response:** changed, now line 198
Line 198. In percentage, round to the nearest whole percent, given the small sample size.

**Authors’ response:** rounded off to 8%, now line 200

Line 198: The table would be clearer to this reviewer with the following editorial changes:
Label the first column “Characteristic,” the second column “Category”, the third column “Number of participants” and fourth column “Percentage or mean.” Then divide the table into three panels, with each panel labeled a centered heading. The first subpanel might be labeled “CHPs (n=42).” the second “In-charges (n=14)”, and the third “Community members (n=76)”. In the data in the last column, use a percent sign for the percentage entries to distinguish them from averages.
I think that the “Summary of data collected” could be added to the text for any important items not already stated, as it does not fit the format above.

**Authors’ response:** this is a great suggestion, and we have structured it as suggested with the summary of data collected as a paragraph following the table (line 202-205).

Line 241. I think it better to describe the rate of 21.1% as the “positivity rate.” By contrast, I would reserve “prevalence” for the results of a population-based testing. In this study, all persons tested likely had a symptom or suspicion of possible malaria. Also change “>” to “>” to clarify that those age 5 were in the upper category.

**Authors’ response:** agreed and edited, now line 244, 249 and 251
Line 250. The table would be strengthened by adding the following additional columns: Estimated pop.<5 yrs, Est. pop > 5 yrs, % of pop <5 tested, % of pop >5 tested. These columns would give useful population-based data. The supplement could indicate how the authors used existing census information and updates to estimate the populations.

**Authors’ response** Thank you for this insightful suggestion. We agree that adding population-based columns would strengthen interpretation of testing coverage by offering an indication of the utilization of facilities, and enhance public health relevance of the findings.

However, in our Kenyan context , population estimates are derived from the Kenya National Bureau of Statistics census data, which are structured according to administrative units (e.g. Counties, Sub-counties, Wards) rather than health facility catchment areas. Primary health facility catchment areas are not formally defined and are highly dynamic. As such, including accurate health facility specific population-based data columns would require strong assumptions that could introduce misclassification and bias. These include assumptions that:

- Clients in the catchment area would all visit the facility linked to their catchment area. This may be dependent on various factors affecting access including geographical distance, socio-economic abilities, insurance coverage among others
- Administrative units are not equal to facility catchment areas/populations i.e. multiple facilities may be included in one ward unit or one facility may be covering populations in multiple wards

In light of this we have not added the column with population-based data at health facility level to avoid potentially misleading estimates. We remain open to suggestions on specific indicators that you think would enrich the interpretation of the data in this table.

Line 345. Those with negative test results referred to health facilities. It would seem helpful if CHPs could distinguish those with negative tests between severe or at risk (based on symptoms, age and pregnancy) and not at risk. Those not at risk might have self-limiting illnesses. The authors might comment on the appropriateness of the guidelines in S2 in view of the resource constraints.

Diagnosis and treatment of other illnesses is beyond the scope of the CHP, they are not able to distinguish what would be self-limiting and what would increase in severity and therefore they immediately refer all cases that are not uncomplicated malaria to the health facility where the facility based health workers have more training, resources for diagnostics, experience and also legal jurisdiction to diagnose and treat a wider range of illnesses. For clarification, this would include:

- Clients with symptoms of malaria that test negative
- Clients with symptoms of complicated malaria
- Pregnant clients with symptoms of malaria or other illness
- Children under 5 years of age with fever or symptoms of malaria or other illness
- Clients with other symptoms for other illnesses

Line 508. The overall prevalence … was 21.1%. This reviewer disagrees with the interpretation. The statistic is the positivity rate. It depends on the relationship between malaria and other conditions giving sufficient suspicion of malaria to warrant a test.

**Authors’ response:** agreed and edited, now line 511
Line 513. Capitalize all words in the title: “World Malaria Report”

**Authors’ response:** agreed and edited, now line 516
Line 516. Please clarify the numerator and denominator of the 17% statistic and present findings from control sites to guide readers in the interpretation.

**Authors’ response:** Within the 10 health facilities where CCMm was being piloted, there were 7,568 confirmed malaria cases, of which 1,268 (17%) were diagnosed and treated by CHPs

This has also been updated in the text, now line 519

Line 547. Add apostrophe: “…CHPs’ abilities…”

**Authors’ response: added, now line 550**

Figure Resubmissions:

While revising your submission, we strongly recommend that you use PLOS’s NAAS tool (<https://ngplosjournals.pagemajik.ai/artanalysis>) to test your figure files. NAAS can convert your figure files to the TIFF file type and meet basic requirements (such as print size, resolution), or provide you with a report on issues that do not meet our requirements and that NAAS cannot fix.

**Authors’ response**: NAAS tool used in the resubmission of all figures
