# Supplementary material for: Implementing community case management of malaria: Stakeholder insights on advancing equitable access in Kilifi County
Source: PLOS Glob Public Health. 2026 Jul 6;6(7):e0006478. doi: 10.1371/journal.pgph.0006478 (PMC13336154; doi:10.1371/journal.pgph.0006478)
Supplement: Peer review history

# Peer review: Decision Letter 0

**DOI:** 10.1371/journal.pgph.0006478.r001

**Authors:** Louisa Messenger

3 Mar 2026

PGPH-D-25-03246

Implementing community case management of malaria: stakeholder insights on advancing equitable access in Kilifi County.

PLOS Global Public Health

Dear Dr. Waweru,

Thank you for submitting your manuscript to PLOS Global Public Health. After careful consideration, we feel that it has merit but does not fully meet PLOS Global Public Health’s publication criteria as it currently stands. Therefore, we invite you to submit a revised version of the manuscript that addresses the points raised during the review process.

Please submit your revised manuscript by Apr 17 2026 11:59PM. If you will need more time than this to complete your revisions, please reply to this message or contact the journal office at globalpubhealth@plos.org. When you're ready to submit your revision, log on to https://www.editorialmanager.com/pgph/ and select the 'Submissions Needing Revision' folder to locate your manuscript file.

Please include the following items when submitting your revised manuscript:

- A letter that responds to each point raised by the editor and reviewer(s). You should upload this letter as a separate file labeled 'Response to Reviewers'.
- A marked-up copy of your manuscript that highlights changes made to the original version. You should upload this as a separate file labeled 'Revised Manuscript with Track Changes'.
- An unmarked version of your revised paper without tracked changes. You should upload this as a separate file labeled 'Manuscript'.

Guidelines for resubmitting your figure files are available below the reviewer comments at the end of this letter.

We look forward to receiving your revised manuscript.

Kind regards,

Louisa Alexandra Messenger, MSc, PhD

Academic Editor

PLOS Global Public Health

Journal Requirements:

1. Please provide a detailed online Financial Disclosure statement. This is published with the article. It must therefore be completed in full sentences and contain the exact wording you wish to be published.

a) State the initials, alongside each funding source, of each author to receive each grant. For example: “This work was supported by the National Institutes of Health (####### to AM; ###### to CJ) and the National Science Foundation (###### to AM).”

b) State what role the funders took in the study. If the funders had no role in your study, please state: “The funders had no role in study design, data collection and analysis, decision to publish, or preparation of the manuscript.”

For more information, please go to our submission guidelines:

https://journals.plos.org/globalpublichealth/s/submission-guidelines#loc-financial-disclosure-statement

2. Please ensure that the funders and grant numbers match between the Financial Disclosure field and the Funding Information tab in your submission form. Note that the funders must be provided in the same order in both places as well.

3. Please update your online Competing Interests statement. If you have no competing interests to declare, please state: "The authors have declared that no competing interests exist."

4. We note that you have indicated that there are restrictions to data sharing for this study. For studies involving human research participant data or other sensitive data, we encourage authors to share de-identified or anonymized data. However, when data cannot be publicly shared for ethical reasons, we allow authors to make their data sets available upon request. For information on unacceptable data access restrictions, please see http://journals.plos.org/globalpublichealth/s/data-availability#loc-unacceptable-data-access-restrictions.

Before we proceed with your manuscript, please address the following prompts:

a) If there are ethical or legal restrictions on sharing a de-identified data set, please explain them in detail (e.g., data contain potentially identifying or sensitive patient information, data are owned by a third-party organization, etc.) and who has imposed them (e.g., a Research Ethics Committee or Institutional Review Board, etc.). Please also provide contact information for a data access committee, ethics committee, or other institutional body to which data requests may be sent.

b) If there are no restrictions, please upload the minimal anonymized data set necessary to replicate your study findings to a stable, public repository and provide us with the relevant URLs, DOIs, or accession numbers. Please see http://www.bmj.com/content/340/bmj.c181.long for guidelines on how to de-identify and prepare clinical data for publication. For a list of recommended repositories, please see https://journals.plos.org/globalpublichealth/s/recommended-repositories. You also have the option of uploading the data as Supporting Information files, but we would recommend depositing data directly to a data repository if possible.

Please update your Data Availability statement in the submission form accordingly."

5. Please upload your main article file as a .doc, .docx or .rtf file.

6. Some material included in your submission may be copyrighted. According to PLOS’s copyright policy, authors who use figures or other material (e.g., graphics, clipart, maps) from another author or copyright holder must demonstrate or obtain permission to publish this material under the Creative Commons Attribution 4.0 International (CC BY 4.0) License used by PLOS journals. Please closely review the details of PLOS’s copyright requirements here: PLOS Licenses and Copyright. If you need to request permissions from a copyright holder, you may use PLOS's Copyright Content Permission form.

Please respond directly to this email or email the journal office and provide any known details concerning your material's license terms and permissions required for reuse, even if you have not yet obtained copyright permissions or are unsure of your material's copyright compatibility.

Potential Copyright Issues:

Figure 1: please (a) provide a direct link to the base layer of the map (i.e., the country or region border shape) and ensure this is also included in the figure legend; and (b) provide a link to the terms of use / license information for the base layer image or shapefile. We cannot publish proprietary or copyrighted maps (e.g. Google Maps, Mapquest) and the terms of use for your map base layer must be compatible with our CC-BY 4.0 license.

Note: if you created the map in a software program like R or ArcGIS, please locate and indicate the source of the basemap shapefile onto which data has been plotted.

If your map was obtained from a copyrighted source please amend the figure so that the base map used is from an openly available source. Alternatively, please provide explicit written permission from the copyright holder granting you the right to publish the material under our CC-BY 4.0 license.

Please note that the following CC BY licenses are compatible with PLOS license: CC BY 4.0, CC BY 2.0 and CC BY 3.0, meanwhile such licenses as CC BY-ND 3.0 and others are not compatible due to additional restrictions.

If you are unsure whether you can use a map or not, please do reach out and we will be able to help you. The following websites are good examples of where you can source open access or public domain maps:

* U.S. Geological Survey (USGS) - All maps are in the public domain. (http://www.usgs.gov)

* PlaniGlobe - All maps are published under a Creative Commons license so please cite “PlaniGlobe, http://www.planiglobe.com, CC BY 2.0” in the image credit after the caption. (http://www.planiglobe.com/?lang=enl)

* Natural Earth - All maps are public domain. (http://www.naturalearthdata.com/about/terms-of-use/)

7. If the reviewer comments include a recommendation to cite specific previously published works, please review and evaluate these publications to determine whether they are relevant and should be cited. There is no requirement to cite these works unless the editor has indicated otherwise.

Additional Editor Comments (if provided):

[Note: HTML markup is below. Please do not edit.]

Reviewers' comments:

Reviewer's Responses to Questions

**Comments to the Author**

1. Does this manuscript meet PLOS Global Public Health’s publication criteria? Is the manuscript technically sound, and do the data support the conclusions? The manuscript must describe methodologically and ethically rigorous research with conclusions that are appropriately drawn based on the data presented.

Reviewer #1: Yes

**********

2. Has the statistical analysis been performed appropriately and rigorously?

Reviewer #1: N/A

**********

3. Have the authors made all data underlying the findings in their manuscript fully available (please refer to the Data Availability Statement at the start of the manuscript PDF file)?

The PLOS Data policy requires authors to make all data underlying the findings described in their manuscript fully available without restriction, with rare exception. The data should be provided as part of the manuscript or its supporting information, or deposited to a public repository. For example, in addition to summary statistics, the data points behind means, medians and variance measures should be available. If there are restrictions on publicly sharing data—e.g. participant privacy or use of data from a third party—those must be specified.

Reviewer #1: Yes

**********

4. Is the manuscript presented in an intelligible fashion and written in standard English?

PLOS Global Public Health does not copyedit accepted manuscripts, so the language in submitted articles must be clear, correct, and unambiguous. Any typographical or grammatical errors should be corrected at revision, so please note any specific errors here.

Reviewer #1: Yes

**********

5. Review Comments to the Author

Please use the space provided to explain your answers to the questions above. You may also include additional comments for the author, including concerns about dual publication, research ethics, or publication ethics. (Please upload your review as an attachment if it exceeds 20,000 characters)

Reviewer #1: Review of Waweru et al.

General comments

This reviewer commends the authors for a thoughtfully conceived and carefully implemented study. I particularly liked the conceptual framework, which articulated the assumptions (often not realized) that might have created a well-functioning CHP program. This reviewer also appreciated the limitations section, which appropriately noted that the program operated under time-limited external funding, which was not generalizable.

This reviewer would encourage the authors to elaborate in the discussion section on how the findings from their study could be useful in the context of most health facilities in Kenya. Resources are often inadequate. Regular health staff are human beings with their strengths, limitations, transportation constraints, and outside responsibilities.

While the health facility data could not be linked directly to the CHPs’ data, could a comparative analysis between the 10 treatment and the 4 control sites indicate whether and by how much the CHPs reduced the workload on health facilities and the resulting savings.

My main recommendation is that the authors suggest ways that the CHP program might be adapted to deliver as many benefits as possible within the resources that are actually available in a typical control facility. Could the system be designed to recognize that remuneration for CHWs is often inadequate and sometimes unavailable? Could CHPs be given non-monetary benefits that could always be guaranteed? Examples include certificates they could put in their kit or display in their house, a pin they could affix to their clothing while working, and priority for being seen when they attend the base primary care facility. For allowances (such as stipends and airtime), could the best CHPs be prioritized. Priorities could be based on activity reports and, perhaps, feedback from their clients who attend health facilities.

Knowing that CHPs and formal health facilities may have inadequate supplies of tests, medications, and staff, could CHWs be taught how to be careful stewards of these scarce resources. Thus, they might be taught to base their decision about whom to test for suspected malaria based on risk (age and symptoms). Similarly, they might be taught for patients who test negative for malaria to offer paracetamol and similarly decide whether to refer patients to a health facility immediately based on risk (age and symptoms). Those not referred immediately should be given clear instructions regarding how long to wait and signs to look for to decide whether they should visit a health facility. For example, if the illness proved to be a bacterial infection, antibiotic treatment would be effective. If it were a viral illness, a treatment such as paracetamol (which a CHP could provide) mght be the only option. If the illness were a symptom of some other disease, the diagnostic skills of the health center staff, and potentially further referrals, would be helpful.

Likewise, the CHPs might focus more efforts on measures that do not require new resources, such as reinforcing recommendations on malaria prevention. These might be geared primarily towards pregnant women and young children, who are most vulnerable to adverse outcomes from malaria. This might entail observing malaria nets, asking about recent use, and refreshing instructions for high-risk community members (e.g. pregnant women and young children).

Finally, suppose the rural health facilities could obtain limited additional resources (e.g. 10% more). The authors would do a service by indicating which malaria-related services would be the highest priority, the expected improvements in health outcome (quantified if possible) and the reasons behind their priorities.

Specific comments

Line 51: It would be helpful if the authors could update the global number to the latest available data.

Line 54: I would suggest writing Malaria in lower case

Line 56: Could you consider rewording “Lack of cooperation from the communities” as it reads like a version of “blame the victim.”

Line 137: Change “The study…” to “The study’s…”

Line 195: Change “majority of…” to “the majority of…”

Line 198. In percentage, round to the nearest whole percent, given the small sample size.

Line 198: The table would be clearer to this reviewer with the following editorial changes:

Label the first column “Characteristic,” the second column “Category”, the third column “Number of participants” and fourth column “Percentage or mean.” Then divide the table into three panels, with each panel labeled a centered heading. The first subpanel might be labeled “CHPs (n=42).” the second “In-charges (n=14)”, and the third “Community members (n=76)”. In the data in the last column, use a percent sign for the percentage entries to distinguish them from averages.

I think that the “Summary of data collected” could be added to the text for any important items not already stated, as it does not fit the format above.

Line 241. I think it better to describe the rate of 21.1% as the “positivity rate.” By contrast, I would reserve “prevalence” for the results of a population-based testing. In this study, all persons tested likely had a symptom or suspicion of possible malaria. Also change “>” to “>” to clarify that those age 5 were in the upper category.

Line 250. The table would be strengthened by adding the following additional columns: Estimated pop.<5 yrs, Est. pop > 5 yrs, % of pop <5 tested, % of pop >5 tested. These columns would give useful population-based data. The supplement could indicate how the authors used existing census information and updates to estimate the populations.

Line 345. Those with negative test results were referred to health facilities. It would seem helpful if CHPs could distinguish those with negative tests between severe or at risk (based on symptoms, age and pregnancy) and not at risk. Those not at risk might have self-limiting illnesses. The authors might comment on the appropriateness of the guidelines in S2 in view of the resource constraints.

Line 508. The overall prevalence … was 21.1%. This reviewer disagrees with the interpretation. The statistic is the positivity rate. It depends on the relationship between malaria and other conditions giving sufficient suspicion of malaria to warrant a test.

Line 513. Capitalize all words in the title: “World Malaria Report”

Line 516. Please clarify the numerator and denominator of the 17% statistic and present findings from control sites to guide readers in the interpretation.

Line 547. Add apostrophe: “…CHPs’ abilities…”

**********

6. PLOS authors have the option to publish the peer review history of their article (what does this mean?). If published, this will include your full peer review and any attached files.

**Do you want your identity to be public for this peer review?**  If you choose “no”, your identity will remain anonymous but your review may still be made public.

For information about this choice, including consent withdrawal, please see our Privacy Policy.

Reviewer #1: **Yes:**  Donald S Shepard

**********

[NOTE: If reviewer comments were submitted as an attachment file, they will be attached to this email and accessible via the submission site. Please log into your account, locate the manuscript record, and check for the action link "View Attachments". If this link does not appear, there are no attachment files.]

-->Figure Resubmissions:

While revising your submission, we strongly recommend that you use PLOS’s NAAS tool (https://ngplosjournals.pagemajik.ai/artanalysis) to test your figure files. NAAS can convert your figure files to the TIFF file type and meet basic requirements (such as print size, resolution), or provide you with a report on issues that do not meet our requirements and that NAAS cannot fix.

After uploading your figures to PLOS’s NAAS tool - https://ngplosjournals.pagemajik.ai/artanalysis, NAAS will process the files provided and display the results in the "Uploaded Files" section of the page as the processing is complete. If the uploaded figures meet our requirements (or NAAS is able to fix the files to meet our requirements), the figure will be marked as "fixed" above. If NAAS is unable to fix the files, a red "failed" label will appear above. When NAAS has confirmed that the figure files meet our requirements, please download the file via the download option, and include these NAAS processed figure files when submitting your revised manuscript.

Attachment Submitted filename: Waweru et al-2025-review01.docx

# Author comment: Author response to Decision Letter 1

**DOI:** 10.1371/journal.pgph.0006478.r002

30 Apr 2026

Attachment Submitted filename: Response to editorial and reviewer comments PGPH-D-25-03246.docx

# Editor report: Decision Letter 1

**DOI:** 10.1371/journal.pgph.0006478.r003

**Authors:** Louisa Messenger, Louisa Messenger

26 May 2026

Implementing community case management of malaria: stakeholder insights on advancing equitable access in Kilifi County.

PGPH-D-25-03246R1

Dear Mrs. Waweru,

We are pleased to inform you that your manuscript 'Implementing community case management of malaria: stakeholder insights on advancing equitable access in Kilifi County.' has been provisionally accepted for publication in PLOS Global Public Health.

Before your manuscript can be formally accepted you will need to complete some formatting changes, which you will receive in a follow up email. A member of our team will be in touch with a set of requests.

Please note that your manuscript will not be scheduled for publication until you have made the required changes, so a swift response is appreciated.

IMPORTANT: The editorial review process is now complete. PLOS will only permit corrections to spelling, formatting or significant scientific errors from this point onwards. Requests for major changes, or any which affect the scientific understanding of your work, will cause delays to the publication date of your manuscript.

If your institution or institutions have a press office, please notify them about your upcoming paper to help maximize its impact. If they'll be preparing press materials, please inform our press team as soon as possible -- no later than 48 hours after receiving the formal acceptance. Your manuscript will remain under strict press embargo until 2 pm Eastern Time on the date of publication. For more information, please contact globalpubhealth@plos.org.

Thank you again for supporting Open Access publishing; we are looking forward to publishing your work in PLOS Global Public Health.

Best regards,

Louisa Alexandra Messenger, MSc, PhD

Academic Editor

PLOS Global Public Health

***********************************************************

Reviewer Comments (if any, and for reference):
